# A novel role for KIFC1-MYH9 interaction in triple-negative breast cancer aggressiveness and racial disparity

**DOI:** 10.1186/s12964-024-01664-0

**Published:** 2024-06-06

**Authors:** Chakravarthy Garlapati, Shriya Joshi, Chunhua Yang, Darshan Shimoga Chandrashekar, Padmashree Rida, Ritu Aneja

**Affiliations:** 1 Department of Biology, Georgia State University, Atlanta, GA 30303 USA; 2 Alkermes Inc, Waltham, MA 02451 USA; 3 Department of Pathology, University of Alabama at Birmingham, Birmingham, AL 35233 USA; 4 Novazoi Theranostics, Salt Lake City, UT 84105 USA; 5 Department of Nutrition Sciences, School of Health Professions, University of Alabama at Birmingham, Birmingham, AL 35233 USA; 6 Small molecule drug discovery, Bristol Myers Squibb, Cambridge, MA 02141 USA; 7 Institute of Biomedical Sciences, Georgia State University, Atlanta, GA 30303 USA

## Abstract

African American (AA) women are twice as likely to develop triple-negative breast cancer (TNBC) as women of European descent. Additionally, AA women with TNBC present a much more aggressive disease course than their European American (EA) counterparts. Thus, there is an unmet clinical need to identify race-specific biomarkers and improve survival outcomes in AA patients with TNBC. The minus-end directed microtubule motor protein kinesin family member C1 (KIFC1) promotes centrosome clustering and chromosomal instability and is often overexpressed in TNBC. Previous findings suggest that KIFC1 plays a role in cell proliferation and migration in TNBC cells from AAs and that the levels of nuclear KIFC1 (nKIFC1) are particularly high in AA patients with TNBC. The nuclear localization of KIFC1 in interphase may underlie its previously unrecognized race-specific association. In this study, we found that in TNBC cells derived from AAs, nKIFC1 interacted with the tumor suppressor myosin heavy chain 9 (MYH9) over EA cells. Treatment of AA TNBC cells with commercial inhibitors of KIFC1 and MYH9 disrupted the interaction between KIFC1 and MYH9. To characterize the racial differences in the KIFC1-MYH9-MYC axis in TNBC, we established homozygous KIFC1 knockout (KO) TNBC cell lines. KIFC1 KO significantly inhibited proliferation, migration, and invasion in AA TNBC cells but not in EA TNBC cells. RNA sequencing analysis showed significant downregulation of genes involved in cell migration, invasion, and metastasis upon KIFC1 KO in TNBC cell lines from AAs compared to those from EAs. These data indicate that mechanistically, the role of nKIFC1 in driving TNBC progression and metastasis is stronger in AA patients than in EA patients, and that KIFC1 may be a critical therapeutic target for AA patients with TNBC.

**Graphical abstract:**

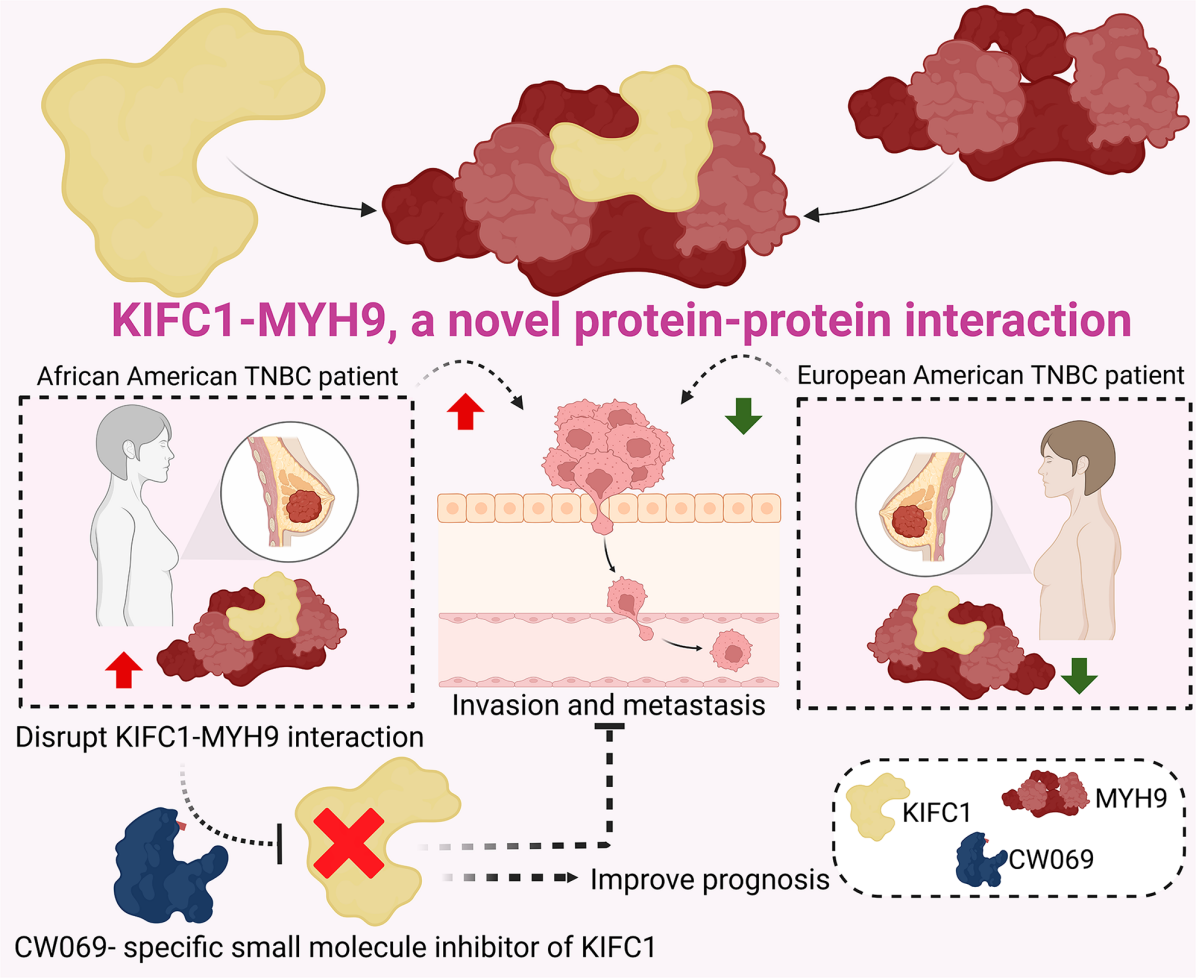

**Supplementary Information:**

The online version contains supplementary material available at 10.1186/s12964-024-01664-0.

## Introduction

African American (AA) patients with breast cancer (BC) experience higher rates of distant metastasis and mortality than European American (EA) patients with BC [1]. The etiology of this disparity is multifactorial [2]. Age-adjusted BC mortality is similar between AA women and non-Latin Caribbean women, who are predominantly of African ancestry, despite significant differences in income and healthcare capacity between these regions [3], suggesting a biological and ancestral component of the disparity.

Triple-negative breast cancer (TNBC) is an aggressive subtype of BC that exhibits poor differentiation, high mitotic activity, high intratumoral heterogeneity, central necrosis, and high rates of distant metastasis, all of which lead to poor prognosis [4]. Nevertheless, not all patients with TNBC experience poor outcomes. Approximately 22–65% of patients with TNBC experience a pathologic complete response (pCR) after neoadjuvant chemotherapy, with 5-year survival rates of ≥ 90% [5, 6]. The prevalence of the TNBC subtype is 3.6 times higher in AA women with BC than in EA women with BC [4]. In addition, tumor stage and grade tend to be higher in AA women with BC than in EA women, whereas age at diagnosis and socioeconomic status tend to be lower in AA women [1]. Even after adjusting for tumor stage, grade, age at diagnosis, and socioeconomic status, AA women have ~ 2-fold higher odds of being diagnosed with TNBC [1], which may partly explain their worse outcomes. Moreover, TNBC predisposition has deep roots in biogeographic ancestry. For instance, approximately 50% of Nigerian [7] and Malian [8] women and 80% of Ghanaian [9] women with BC have TNBC. In contrast, TNBC accounts for approximately 15% of BC cases in EAs [9, 10] and in white British women [7, 11]. However, the genetic variants that link African ancestry to TNBC predisposition are unknown. The identification of prognostic and predictive biomarkers may provide valuable information for TNBC.

There is no standard chemotherapeutic regimen for TNBC; commonly used treatments include taxanes and anthracyclines [12]. The pCR can be increased to approximately 20% by adding carboplatin to a weekly taxane/anthracycline backbone [13]. Before treatment with systemic adjuvant therapy, patients with TNBC are stratified based on tumor stage, with no consideration given to race, ethnicity, or the underlying molecular cause of cancer. Moreover, there are currently no approved prognostic assays or targeted therapies for TNBC. Only 5–10% of patients with BC in clinical trials are AAs, and very few studies have stratified outcomes by ethnicity; thus, there is limited information on chemotherapy response for AA patients with TNBC [3]. Consequently, high-risk AA patients with TNBC may not be identified using standard screening tools and may not be administered effective treatment. Additionally, current treatments may be less effective for AAs with TNBC because they are underrepresented in clinical trials. The development of personalized medicine interventions for AAs has been marginal because the AA population is highly admixed, which dilutes the effect of African ancestry in analyses of self-reported race, tumor biology, and clinical outcomes. A critical barrier to progress in customizing treatments and improving disease outcomes in AA patients with TNBC is the lack of therapeutically actionable biomarkers of disease aggressiveness in this patient population.

We have previously shown that KIFC1 is a prognostic biomarker and an emerging therapeutic target in cancer, particularly TNBC. Unlike nonmalignant cells, cancer cells often possess extra centrosomes. Supernumerary centrosomes are considered a hallmark of BC [14, 15]. During mitosis, KIFC1 clusters supernumerary centrosomes in cancer cells by crosslinking and sliding microtubules [16]. Centrosome clustering allows cancer cells to avoid mitotic spindle multipolarity, which can result in cell death by mitotic catastrophe or multipolar division. We validated KIFC1 as a therapeutic target in AA patients with TNBC.

## Materials and methods

### Cell culture

MDA-MB-468 (AA), HCC1806 (AA), MDA-MB-231 (EA), and BT-549 (EA) cells were grown in L15 or RPMI-1640 media supplemented with appropriate growth factors and fetal bovine serum, according to ATCC guidelines. Cells were maintained in a humidified 5% CO_2_ atmosphere at 37 °C.

### Immunoprecipitation LC-MS (IP-MS)

Immunoprecipitation (IP) was performed using a Pierce Crosslink Magnetic IP/Co-IP Kit (Thermo Fisher Scientific, #88,805). Anti-KIFC1 antibody (2.5 µg; Abcam, #ab172620) and rabbit monoclonal IgG (Abcam, #ab172730) were used to crosslink the beads. Nuclear lysates were used for manual antigen immunoprecipitation and elution following the manufacturer’s protocol. IP samples were then processed using in-solution digestion and filtered through a 0.22 μm filter before being subjected to LC-MS-based proteomics analysis. RP-HPLC–MS/MS analysis utilized an LTQ-Orbitrap Elite mass spectrometer (Thermo Fisher) with EASY-spray source and nano-LC UltiMate 3000 high-performance liquid chromatography system (Thermo Fisher). Separation was conducted using EASY-Spray PepMap C18 columns (50 cm; particle size, 2 μm; pore size, 100 Å; Thermo Fisher Scientific) with a linear gradient from 3 to 40% solvent B over 30 min at a flow rate of 300 nL/min (mobile phase A, 2% ACN, 98% H_2_O, 0.1% FA; mobile phase B, 80% ACN, 20% H_2_O, 0.1% FA). The LTQ-Orbitrap Elite mass spectrometer operated in data-dependent mode, conducting a full-scan survey MS experiment (m/z range 375–1500; resolution at m/z 200, 60,000; max ion accumulation time, 50 ms).

For data processing, raw data were converted to mgf files using Proteome Discoverer 1.3 and analyzed with pFind (version 2.1). The human proteome sequence database (Uniprot_swissprot plus Uniprot_TrEMBL) with reversed sequences was used. Mass tolerances were set at 20 ppm for precursor ions and 25 mmu for fragment ions. A 1% FDR was estimated and applied at the peptide-spectrum match level. Mgf data were compared to the human Uniprot database, with static modification of carbamidomethyl (Cys, + 57.0214), dynamic modifications of oxidation (Met, + 15.9949), and acetylation (N-terminal). Trypsin with two allowed missed cleavages was used. pBuild software removed redundant protein entries, and a unique peptide (sequence: IIGLDQVAGMSETALPGAFK, Rt: 24 min) of MYH9 was identified for quantitation and group comparison.”

### Time-resolved foster’s resonance energy transfer (TR-FRET) assay

The nuclear fractions of all cell lines were isolated using the NE-PER Nuclear and Cytoplasmic Extraction Kit (Thermo Fisher Scientific, #78,833). The protein concentration was determined using the Bradford assay. Protein samples (30 µg) were diluted in TR-FRET dilution buffer (50 µL final volume; Thermo Fisher Scientific, #PV3574) and added in triplicates in 96-well black clear bottom plates. The nuclear fractions were incubated with CW069, blebbistatin, and their combination for 1 h at 200 revolutions per minute (RPM). Untreated nuclear fractions were used as controls. APC (Abcam, #ab201807) or PE lightning-link conjugation kits (Abcam, #ab102918) were used for antibody tagging, according to the manufacturer’s guidelines. Cell lysates (treated or untreated) were incubated with KIFC1-APC and MYH9-PE tagged antibodies (1:200 dilution) individually and in combination for 1 h at 200 RPM. Samples without antibodies were used to subtract background noise. After incubation, fluorescence spectra were recorded at 488 nm excitation and 575 nm and 675 nm emission using a SpectraMax MD-M2 plate reader. Data were analyzed using GraphPad Prism 10.0 (Dotmatics).

### Guide RNA design

CRISPR guide RNAs (gRNAs) were designed using the online tool https://portals.broadinstitute.org/gppx/crispick/public/#/. gRNAs were 23–500 nucleotides long and had a high specificity score (> 85). gRNA specificity was assessed using the CRISPR RGEN tool Cas-OFFinder http://www.rgenome.net/cas-offinder/; gRNAs with 1 or 2 mismatches were avoided. Oligos for pSpCas9(BB)-2A-Puro (PX459) V2.0 (Addgene plasmid, #62988) and reverse complement oligos (Sigma-Aldrich) containing 5’ and 3’ overhangs were used for cloning. The sequences of the gRNA oligos are shown below:

1. HSET-sgRNA1-FP: 5’-CACCGTCCCCCCTATTGGAAGTAAA-3’.
2. HSET-sgRNA1-RP: 5’-AAACTTTACTTCCAATAGGGGGGAC-3’.
3. HSET-sgRNA2-FP: 5’-CACCGCAGGAAGCAGACTCAAGAGG-3’.
4. HSET-sgRNA2-RP: 5’-AAACCCTCTTGAGTCTGCTTCCTGC-3’.

### Cloning

The plasmid pSpCas9(BB)2APuro (PX459) V2.0 (Addgene plasmid, #62,988) was digested with BbsI endonuclease (NEB, #R0539S/R0539L). The BbsI-digested PX459 vector was purified using a QIAquick Gel Extraction Kit (QIAGEN, #28,704/28,706). The annealed oligo duplex was ligated into the digested PX459 vector, followed by transformation of the ligation product into Stbl3 competent cells (Thermo Fischer Scientific, #C737303). The recombinant plasmid was isolated using the QIAprep Spin Miniprep kit (Qiagen, #27,106), and sequencing was performed to confirm cloning success.

### Isolation of CRISPR knockout clonal cells

The cells were transfected with the recombinant plasmid using Lipofectamine LTX (Thermo Fischer Scientific, #15,338,100) and incubated overnight at 37 °C. The culture medium was replaced with fresh growth media containing puromycin to select transfected cells. Live cells (1 cell per well) were seeded into 96-well plates containing 100 µL/well growth medium and were incubated at 37 °C. After 2–3 weeks, cell colonies were screened by genomic sequencing to confirm the knockout (KO). Western blotting was performed to identify homogeneous KO clones.

### Gene set enrichment and modeling of gene interaction networks

Differentially regulated genes from the RNA-seq data were imported to the Ingenuity Pathways Analysis (IPA) software (Ingenuity Systems; Qiagen, Redwood City, CA, USA) www.ingenuity.com/) and were subjected to network and upstream regulation analysis (URA) to analyze upstream molecules. These analyses can connect the genes in the dataset and the Ingenuity knowledge base (genes only) with direct or indirect relationships based on changes in expression. For all the analyses, experimental log ratio from − 3.0 (down) to 3.0 (up) and *P*-value > 0.05 were used as cutoffs. To understand the activation/inhibition of differentially expressed (DE) genes, a mechanistic networks (MN) analysis was performed using IPA. MN analysis connects the upstream regulators with the signaling cascades to explain the observed changes in differential gene expression, with a p-value cutoff of 0.01. The predicted activation/inhibition and activation Z scores were derived from MN analysis. Pathway analysis identified the relation of genes with various canonical pathways. Pathway analysis provided the percentage of genes from the dataset to the total number of genes in a specific pathway. Biological processes (disease) and functions of various genes affected by the deregulation were identified and connected using downstream effector analysis (DEA). DEA predicted gene activation state, calculates Z score, p-value of overlap. By using precise algorithms, IPA predicted functional regulatory networks. These networks were predicted using gene expression data uploaded by the user. Each network was ranked using a significance score based on the fit of the network to the focus genes in the database. Significance scores represent the negative log of the p-value for the probability of focus genes being found together by chance [17].

### Immunoblotting

Cells at ~ 70% confluence were used to prepare the protein samples. Cells were lysed in RIPA lysis buffer supplemented with a protease inhibitor cocktail. Polyacrylamide gel electrophoresis was performed to resolve proteins, which were transferred onto polyvinylidene fluoride membranes (Millipore). The Pierce ECL chemiluminescence detection kit (Thermo Fisher Scientific) was used to visualize the protein bands, and β-actin and HDAC-2 (Abcam, #ab32117) were used as loading controls.

### Cell proliferation assay

A BrdU cell proliferation kit (EMD Millipore, #2750) was used to assess cell proliferation. KIFC1 KO and control cells were seeded in 96-well plates at a density of ~ 5 × 10^3^ cells per well. Each cell line was seeded in triplicates. Following cell adhesion, cells were incubated with BrdU for 4 h. BrdU incorporation was measured spectrophotometrically at 450 nm using the TMB substrate provided in the kit.

### Immunofluorescence staining

Cells were seeded in 6-well plates, and when cell confluency reached 80%, cells were incubated with BrdU for 24 h at 37 °C. After PBS washes, cells were fixed with formaldehyde for 30 min at − 20 °C. Subsequently, cells were subjected to acid hydrolysis for 30 min, followed by neutralization with borate buffer. Cells were thoroughly washed with PBS, blocked with 5% bovine serum albumin (BSA) containing 0.1% Triton X for 1 h, and incubated with BrdU and α-tubulin antibody at 37 °C for 40–45 min. Cells were then incubated with a cocktail of rabbit and mouse fluorescent secondary antibodies at 37 °C for 40–45 min. Nuclei were counterstained with Hoechst, and coverslips were mounted using ProLong gold antifade. Images acquired under a confocal microscope (LSM 700) were analyzed using ImageJ. To assess the localization of KIFC1 and MYH9, immunofluorescence staining was performed using untreated and treated (CW069, blebbistatin, and their combination) AA and EA TNBC cells.

### Boyden chamber invasion assay

CRISPR KO and control cells were harvested at 70–80% confluence. Cells (10 × 10^4^–20 × 10^4^) suspended in serum-free medium were seeded on inserts with 8 μm pores in 24-well transwell plates (Corning, #3422). All cell lines were seeded in triplicates. Serum-free medium was added to the bottom of the transwell to achieve serum-starved conditions. The plates were incubated for 12–18 h in 5% CO_2_ at 37 °C. Subsequently, the Boyden chambers were fixed with 3.7% paraformaldehyde and stained with 4% crystal violet. Five different fields for each sample were observed, colonies were counted independently by two observers, and the mean colony count was determined. All images were acquired using AxioVision (Carl Zeiss).

### Scratch-wound migration assay

KIFC1 KO and control cells were seeded in 6-well plates. At 70–80% confluency, a wound was scratched gently using a pipette tip. Images were obtained from six optical fields using a Zeiss Primovert phase-contrast microscope. Images were acquired at 0 h and 24 h of incubation, and cell migration was analyzed using Fiji (ImageJ) and Adobe Photoshop (Adobe).

### Xenograft animal model

Nude female mice were used to establish xenograft models following protocols that adhered to the Institutional Animal Care and Use Committee guidelines. To determine the number of animals required for the study, we performed a power analysis using GraphPad Prism 9. HCC1806 (AA) and MDA-MB-231 (EA) cells were subcutaneously injected into the right flank of the mice (4 × 10^6^ cells per flank). When tumors reached 100 mm^3^, mice were divided into two groups (*n* = 12 for each treatment and race group): vehicle and CW069 (40 mg/kg). No animals were excluded from the study at any time point until the termination of the experiment, and all animals were randomized to the treatment groups. CW069 was administered intraperitoneally twice a week for up to 28 days. Tumor growth was measured once per week using Vernier calipers, and body weight was recorded for up to 4 weeks. Tumor volume was calculated as follows: length × (width)^2^ ÷ 2. At the end of the experiment, all mice were euthanized. The investigator was blinded to the race of the cell lines throughout the process (from the injection of cell lines to the completion of all the analyses).

### Statistical analyses

All experiments were performed in triplicate, and data were used to calculate statistical significance using two-tailed unpaired Student’s t-test with Welch’s correction, unpaired nonparametric Mann–Whitney or Kolmogorov–Smirnov test, or one- or two-way analysis of variance (ANOVA) with Tukey’s test for multiple comparisons. Survival data were analyzed using the Mantel–Cox test, and Pearson’s coefficient was used to assess correlations among variables. Data were expressed as mean ± standard error of the mean. Statistical analyses were performed using GraphPad Prism version 9. Two-tailed t-test was used for comparative groups. *P*-values ≤ 0.05 (two-tailed t-test) were considered significant. For IPA analyses, a Z score (− 2.0 ≤ Z ≥ 2.0) was considered significant.

## Results

### KIFC1 binds to MYH9 is higher in AA TNBC cells

**Fig. 1:**
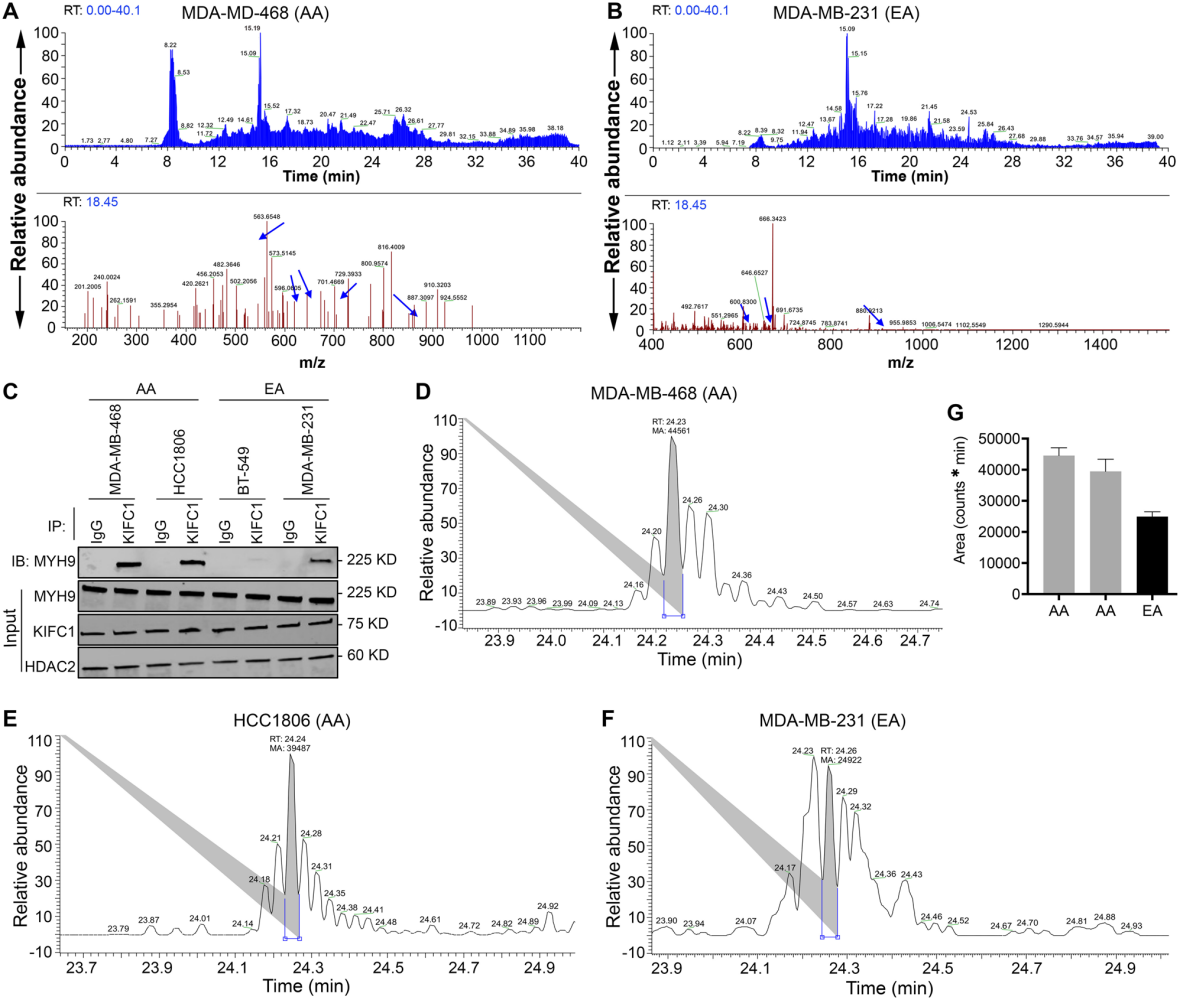
Higher abundance of MYH9 with KIFC1 in AA than in EA TNBC cell lines. (**A**, **B**) LC-MS total ion current (TIC) chromatograms of all peptides in the nKIFC1 immunoprecipitates (IP) of AA (**A**) and EA (**B**) cell lines are shown in the upper panel. The blue arrows in the lower panel represent MYH9 peptides. (**C**) Western blot of KIFC1 IP samples probed for MYH9. (**D**-**G**) Relative abundance of MYH9-specific peptide at 24 min RT in AA (**D**, **E**) and EA (**F**), with respective quantification (**G**)

Previously, we have reported the ability of nKIFC1 to predict disease outcomes in AA patients with TNBC [18]. Identifying the binding partners of KIFC1 in AA TNBC may provide further insights into the role of KIFC1 in TNBC aggressiveness in AA patients. To identify the binding partners of KIFC1, we immunoprecipitated KIFC1 from nuclear extracts of AA and EA TNBC cell lines, and samples were subjected to untargeted mass spectrometry. Our data revealed MYH9 as the top nKIFC1-binding partner in the two AA TNBC cell lines (Fig. 1A). nKIFC1 was also found to be a binding partner of MYH9 in one of the EA TNBC cell lines (MDA-MB-231, Fig. 1B); no interaction between MYH9 and nKIFC1 was detected in the second EA TNBC cell line (BT-549). The relative abundance of MYH9 in nKIFC1 immunoprecipitated in the AA TNBC cell lines MDA-MB-468 and HCC1806 was higher (~ 1.8 fold and ~ 1.6 fold, respectively) than that in the EA TNBC cell line MDA-MB-231 (Fig. 1C–G). Interestingly, β-actin, γ-actin, α-actinin, and DDX helicases, including DDX5 (known to bind RNA Pol II) and DDX17, were detected only in AA TNBC cell lines (Tables 1 and 2; see the Excel workbook for details). However, immunoprecipitated histones were detected in the two TNBC cell lines from EA patients (Tables 3 and 4; see details in the Excel workbook). These data suggest that the association between nKIFC1 and MYH9 in TNBC cells is more prominent in AAs and that the composition of the nKIFC1-Myh9 complex (and potentially its association with chromatin) differs between AA and EA TNBC cells.

**Table 1:** HCC1806 (AA) KIFC1 bound proteins (IP-MS)

Protein ID	Coverage (%)	Spectra	Unique peptides
sp|P35579|MYH9_HUMAN	25.71	48	39
sp|P35908|K22E_HUMAN	23.16	8	8
sp|P0CG48|UBC_HUMAN	21.02	1	1
sp|P0CG47|UBB_HUMAN	20.96	1	1
sp|P60709|ACTB_HUMAN	19.73	4	4
sp|P63261|ACTG_HUMAN	19.73	4	4
sp|P13645|K1C10_HUMAN	15.75	7	6
sp|O00159|MYO1C_HUMAN	14.77	11	11
sp|P08729|K2C7_HUMAN	13.86	5	5
sp|P05787|K2C8_HUMAN	13.04	5	5
sp|P62987|RL40_HUMAN	12.5	1	1
sp|P35527|K1C9_HUMAN	11.88	4	4
sp|P02538|K2C6A_HUMAN	10.82	5	5
sp|P48668|K2C6C_HUMAN	10.82	5	5
sp|P62979|RS27A_HUMAN	10.26	1	1
sp|O43795|MYO1B_HUMAN	9.86	9	9
sp|P47914|RL29_HUMAN	9.43	1	1
sp|P02533|K1C14_HUMAN	8.9	3	3
sp|P35580|MYH10_HUMAN	8.86	12	11
sp|P17844|DDX5_HUMAN	8.31	4	4
sp|O43707|ACTN4_HUMAN	7.57	5	5
sp|P31943|HNRH1_HUMAN	7.57	2	2
sp|Q9BQE3|TBA1C_HUMAN	7.57	2	2
sp|Q9NY65|TBA8_HUMAN	7.57	2	2
sp|Q13748|TBA3C_HUMAN	7.56	2	2
sp|Q6PEY2|TBA3E_HUMAN	7.56	2	2
sp|Q71U36|TBA1A_HUMAN	7.54	2	2
sp|Q7Z406|MYH14_HUMAN	6.97	11	9
sp|P23246|SFPQ_HUMAN	6.93	3	3
sp|Q9BW19|KIFC1_HUMAN	6.09	3	3
sp|P21333|FLNA_HUMAN	6.08	9	9
sp|P04259|K2C6B_HUMAN	6.03	3	3
sp|P12814|ACTN1_HUMAN	5.49	4	4
sp|P13647|K2C5_HUMAN	5.42	3	3
sp|Q13490|BIRC2_HUMAN	5.18	1	1
sp|Q16643|DREB_HUMAN	5.08	2	2
sp|P19012|K1C15_HUMAN	5.04	2	2
sp|P13646|K1C13_HUMAN	5.02	2	2
sp|P61978|HNRPK_HUMAN	4.97	2	2
sp|O43175|SERA_HUMAN	4.88	2	2
sp|P08779|K1C16_HUMAN	4.86	2	2
sp|Q9UM54|MYO6_HUMAN	4.79	4	4
sp|P68366|TBA4A_HUMAN	4.69	1	1
sp|P36578|RL4_HUMAN	4.68	2	2
sp|P68363|TBA1B_HUMAN	4.66	1	1
sp|P0DME0|SETLP_HUMAN	4.64	1	1
sp|Q04695|K1C17_HUMAN	4.63	2	2
sp|Q9UJ72|ANX10_HUMAN	4.63	1	1
sp|O94832|MYO1D_HUMAN	4.57	4	4
sp|Q16630|CPSF6_HUMAN	4.54	1	1
sp|P26368|U2AF2_HUMAN	4.42	1	1
sp|P02545|LMNA_HUMAN	4.37	2	2
sp|P26599|PTBP1_HUMAN	4.33	1	1
sp|P0DMV8|HS71A_HUMAN	4.21	2	2
sp|P0DMV9|HS71B_HUMAN	4.21	2	2
sp|P52597|HNRPF_HUMAN	4.1	1	1
sp|O95678|K2C75_HUMAN	3.99	2	2
sp|Q9UHB6|LIMA1_HUMAN	3.95	2	2
sp|P55795|HNRH2_HUMAN	3.79	1	1
sp|P0CG39|POTEJ_HUMAN	3.76	2	2
sp|Q13561|DCTN2_HUMAN	3.74	1	1
sp|O00515|LAD1_HUMAN	3.68	1	1
sp|A5A3E0|POTEF_HUMAN	3.63	2	2
sp|P0CG38|POTEI_HUMAN	3.63	2	2
sp|Q6S8J3|POTEE_HUMAN	3.63	2	2
sp|Q92841|DDX17_HUMAN	3.57	2	2

**Table 2:** MDA-MB-468 (AA) KIFC1 bound proteins (IP-MS)

Protein ID	Coverage (%)	Spectra	Unique peptides
sp|P35579|MYH9_HUMAN	29.69	56	46
sp|P60709|ACTB_HUMAN	18.67	5	4
sp|P63261|ACTG_HUMAN	18.67	5	4
sp|P21333|FLNA_HUMAN	13.41	21	21
sp|P35580|MYH10_HUMAN	10.27	13	13
sp|P26368|U2AF2_HUMAN	9.05	4	3
sp|O94832|MYO1D_HUMAN	8.75	6	6
sp|Q7Z406|MYH14_HUMAN	8.32	12	11
sp|P04350|TBB4A_HUMAN	8.11	3	3
sp|P07437|TBB5_HUMAN	8.11	3	3
sp|P68371|TBB4B_HUMAN	8.09	3	3
sp|Q13509|TBB3_HUMAN	8	3	3
sp|O00159|MYO1C_HUMAN	7.71	7	6
sp|P04259|K2C6B_HUMAN	7.62	4	4
sp|Q16643|DREB_HUMAN	7.55	4	3
sp|Q9BW19|KIFC1_HUMAN	7.43	2	2
sp|Q16630|CPSF6_HUMAN	7.08	3	2
sp|Q6ZMS4|ZN852_HUMAN	6.26	1	1
sp|O43707|ACTN4_HUMAN	5.93	4	4
sp|Q9UM54|MYO6_HUMAN	5.72	5	5
sp|Q13885|TBB2A_HUMAN	5.39	2	2
sp|Q9BVA1|TBB2B_HUMAN	5.39	2	2
sp|O43795|MYO1B_HUMAN	5.28	4	4
sp|Q01082|SPTB2_HUMAN	4.99	8	7
sp|P61978|HNRPK_HUMAN	4.97	1	1
sp|Q9BQE3|TBA1C_HUMAN	4.45	1	1
sp|Q13748|TBA3C_HUMAN	4.44	1	1
sp|Q6PEY2|TBA3E_HUMAN	4.44	1	1
sp|P68363|TBA1B_HUMAN	4.43	1	1
sp|Q71U36|TBA1A_HUMAN	4.43	1	1
sp|Q9BYX7|ACTBM_HUMAN	4.27	1	1
sp|P63267|ACTH_HUMAN	4.26	1	1
sp|Q562R1|ACTBL_HUMAN	4.26	1	1
sp|P62736|ACTA_HUMAN	4.24	1	1
sp|P68032|ACTC_HUMAN	4.24	1	1
sp|P68133|ACTS_HUMAN	4.24	1	1
sp|P38646|GRP75_HUMAN	4.12	2	2
sp|Q9UHR5|S30BP_HUMAN	3.9	1	1
sp|P26599|PTBP1_HUMAN	3.77	1	1
sp|Q13561|DCTN2_HUMAN	3.74	1	1
sp|Q92841|DDX17_HUMAN	3.7	2	2
sp|Q6S8J3|POTEE_HUMAN	3.63	2	2
sp|P48741|HSP77_HUMAN	3.54	1	1
sp|P35749|MYH11_HUMAN	3.45	6	5
sp|Q9UHB6|LIMA1_HUMAN	3.43	2	2
sp|Q9BUF5|TBB6_HUMAN	3.14	1	1
sp|Q9NR30|DDX21_HUMAN	2.81	2	2
sp|P05783|K1C18_HUMAN	2.79	1	1
sp|O75909|CCNK_HUMAN	2.76	1	1
sp|Q9Y230|RUVB2_HUMAN	2.59	1	1
sp|P02768|ALBU_HUMAN	2.46	3	2
sp|P17844|DDX5_HUMAN	2.44	1	1

**Table 3:** MDA-MB-231 (EA) KIFC1 bound proteins (IP-MS)

Protein ID	Coverage (%)	Spectra	Unique peptides
sp|P04264|K2C1_HUMAN	20.65	14	8
sp|P35527|K1C9_HUMAN	16.69	6	4
sp|P16403|H12_HUMAN	15.96	8	5
sp|P35908|K22E_HUMAN	15.81	10	6
sp|P10412|H14_HUMAN	15.53	8	5
sp|P16402|H13_HUMAN	15.38	8	5
sp|P16401|H15_HUMAN	15.04	8	6
sp|P62750|RL23A_HUMAN	11.54	2	2
sp|P62899|RL31_HUMAN	11.2	3	1
sp|P62753|RS6_HUMAN	10.84	2	2
sp|E9PRG8|CK098_HUMAN	10.66	1	1
sp|P47914|RL29_HUMAN	9.43	1	1
sp|O60814|H2B1K_HUMAN	8.73	1	1
sp|P06899|H2B1J_HUMAN	8.73	1	1
sp|P23527|H2B1O_HUMAN	8.73	1	1
sp|P33778|H2B1B_HUMAN	8.73	1	1
sp|P57053|H2BFS_HUMAN	8.73	1	1
sp|P58876|H2B1D_HUMAN	8.73	1	1
sp|P62807|H2B1C_HUMAN	8.73	1	1
sp|Q16778|H2B2E_HUMAN	8.73	1	1
sp|Q5QNW6|H2B2F_HUMAN	8.73	1	1
sp|Q8N257|H2B3B_HUMAN	8.73	1	1
sp|Q93079|H2B1H_HUMAN	8.73	1	1
sp|Q99877|H2B1N_HUMAN	8.73	1	1
sp|Q99879|H2B1M_HUMAN	8.73	1	1
sp|Q99880|H2B1L_HUMAN	8.73	1	1
sp|Q96A08|H2B1A_HUMAN	8.66	1	1
sp|P62263|RS14_HUMAN	8.61	1	1
sp|P83731|RL24_HUMAN	8.28	2	1
sp|P12273|PIP_HUMAN	8.22	1	1
sp|P02538|K2C6A_HUMAN	8.16	4	4
sp|P48668|K2C6C_HUMAN	8.16	4	4
sp|P04259|K2C6B_HUMAN	8.16	5	4
sp|P13647|K2C5_HUMAN	8.14	4	4
sp|P13645|K1C10_HUMAN	7.71	5	4
sp|P62249|RS16_HUMAN	7.53	1	1
sp|P46776|RL27A_HUMAN	7.43	1	1
sp|Q02543|RL18A_HUMAN	7.39	1	1
sp|P37108|SRP14_HUMAN	7.35	1	1
sp|P60660|MYL6_HUMAN	7.28	1	1
sp|P06748|NPM_HUMAN	7.14	3	1
sp|Q07020|RL18_HUMAN	6.91	1	1
sp|P46778|RL21_HUMAN	6.88	1	1
sp|P35579|MYH9_HUMAN	6.84	15	11
sp|P19388|RPAB1_HUMAN	6.67	1	1
sp|P61353|RL27_HUMAN	6.62	1	1
sp|P62241|RS8_HUMAN	6.25	1	1
sp|P62081|RS7_HUMAN	6.19	1	1
sp|P0C0S5|H2AZ_HUMAN	5.47	1	1
sp|Q71UI9|H2AV_HUMAN	5.47	1	1
sp|Q96KK5|H2A1H_HUMAN	5.47	1	1
sp|Q99878|H2A1J_HUMAN	5.47	1	1

**Table 4:** BT-549 (EA) KIFC1 bound proteins (IP-MS)

Protein ID	Coverage (%)	Spectra	Unique peptides
sp|P16403|H12_HUMAN	15.96	8	4
sp|P10412|H14_HUMAN	15.53	8	4
sp|P16402|H13_HUMAN	15.38	8	4
sp|P62899|RL31_HUMAN	11.2	1	1
sp|Q6NVV1|R13P3_HUMAN	10.78	1	1
sp|P62829|RL23_HUMAN	10.71	2	1
sp|P35908|K22E_HUMAN	9.7	4	4
sp|Q86WX3|AROS_HUMAN	9.56	1	1
sp|P47914|RL29_HUMAN	9.43	1	1
sp|P13056|NR2C1_HUMAN	8.29	1	1
sp|P83731|RL24_HUMAN	8.28	1	1
sp|P04264|K2C1_HUMAN	7.92	5	3
sp|P62913|RL11_HUMAN	7.87	1	1
sp|P62249|RS16_HUMAN	6.85	1	1
sp|P46776|RL27A_HUMAN	6.76	1	1
sp|P61353|RL27_HUMAN	6.62	1	1
sp|P22492|H1T_HUMAN	5.8	5	2
sp|P16401|H15_HUMAN	5.75	1	1
sp|P13645|K1C10_HUMAN	5.65	2	2
sp|Q02539|H11_HUMAN	5.58	5	2
sp|P40429|RL13A_HUMAN	5.42	1	1
sp|P62917|RL8_HUMAN	4.28	1	1
sp|Q9Y2J0|RP3A_HUMAN	4.18	1	1
sp|P04259|K2C6B_HUMAN	3.37	4	2
sp|P22090|RS4Y1_HUMAN	3.04	1	1
sp|P62701|RS4X_HUMAN	3.04	1	1
sp|Q8TD47|RS4Y2_HUMAN	3.04	1	1
sp|Q68DH5|LMBD2_HUMAN	3.02	1	1
sp|P08727|K1C19_HUMAN	2.25	1	1
sp|Q04695|K1C17_HUMAN	2.08	1	1
sp|Q96Q27|ASB2_HUMAN	2.04	1	1
sp|P68104|EF1A1_HUMAN	1.73	1	1
sp|Q05639|EF1A2_HUMAN	1.73	1	1
sp|Q5VTE0|EF1A3_HUMAN	1.73	1	1

### Pharmacological inhibition of KIFC1 and MYH9 disrupts their interaction and nuclear localization in AA TNBC cells

**Fig. 2:**
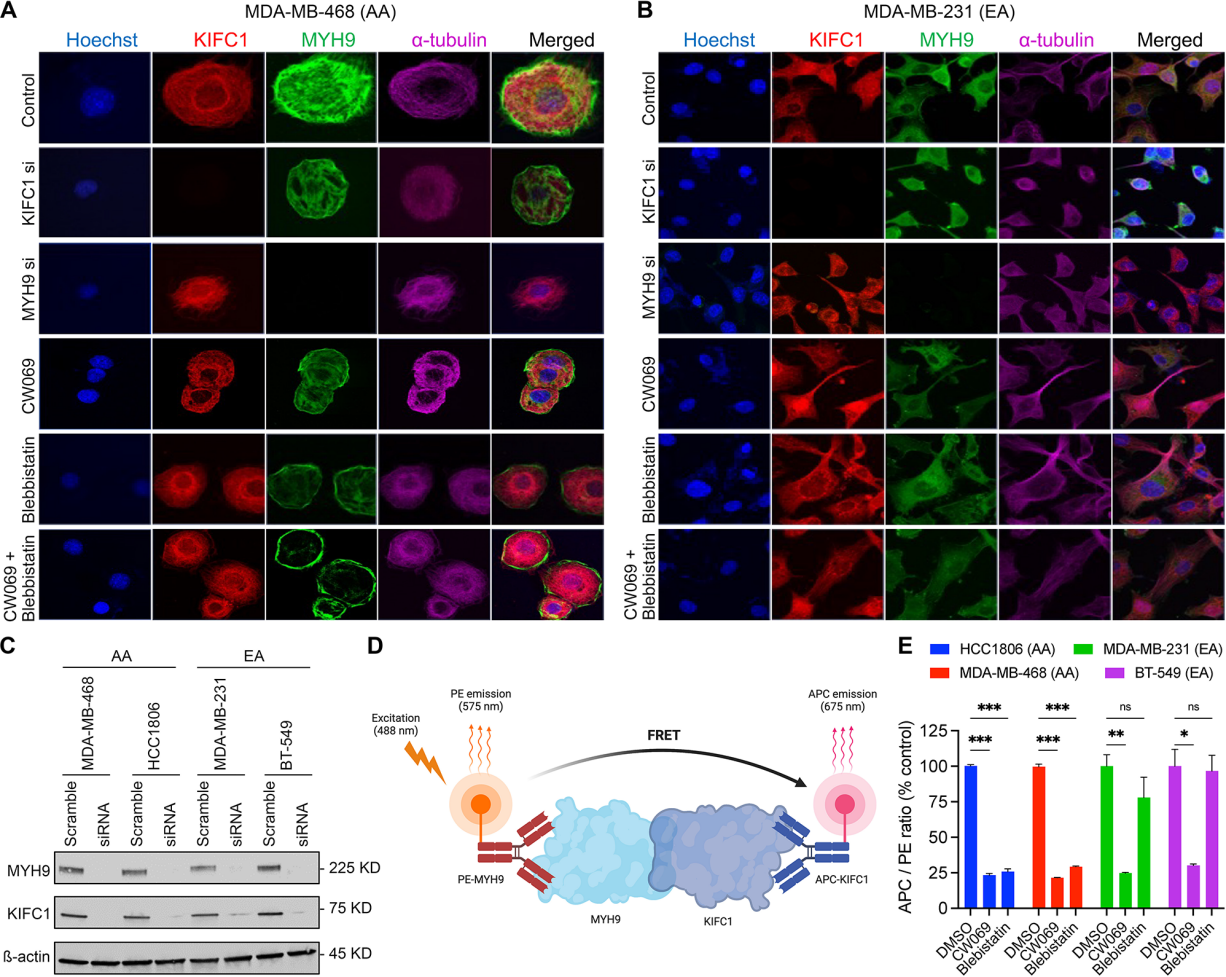
Localization of KIFC1 and MYH9 in AA and EA TNBC cells. Representative IF images (**A, B**) showing localization of KIFC1 and MYH9 in control, KIFC1 si, MYH9 si, CW069, blebbistatin and CW + blebbistatin treatment groups in AA (**A**) and EA (**B**) TNBC cells. (**C**) Western blot showing KIFC1 and MYH9 KD efficiency in AA and EA TNBC cells. Schematic overview of TR-FRET design (**D**) and quantitative graphs (**E**) showing FRET of KIFC1 and MYH9 in AA and EA TNBC cells

Next, we assessed whether the localization of KIFC1 and MYH9 differed in AA and EA TNBC cells. At the basal level and upon treatment with the KIFC1 inhibitor CW069, we did not observe significant differences in the localization of KIFC1 or MYH9 between AA and EA TNBC cells (Fig. 2A, B). However, when the cells were treated with MYH9 inhibitor blebbistatin alone or in combination with CW069, there was a significant difference in MYH9 localization between AA and EA TNBC cells. Specifically, treatment of AA TNBC cells with blebbistatin alone or in combination with CW069 results in a shift in MYH9 localization towards the cell periphery. However, no shifts in localization were observed in EA TNBC cells (Fig. 2B). These results indicate that blebbistatin alone or in combination with CW069 affects MYH9 localization, which could interfere with the KIFC1-MYH9 interaction. KD efficiency upon KIFC1 or MYH9 siRNA treatment is shown in Fig. 2C. Next, we used TR-FRET assay to elucidate the effects of KIFC1 and MYH9 inhibition on KIFC1-MYH9 interaction in AA and EA TNBC cells (Fig. 2D). The significant loss of TR-FRET signal in AA TNBC cells upon treatment with CW069, blebbistatin, or their combination was observed (Fig. 2E). These results indicate that pharmacological inhibition of KIFC1 and MYH9 disrupts their localization and interaction, particularly in AA TNBC cells.

**Fig. 3:**
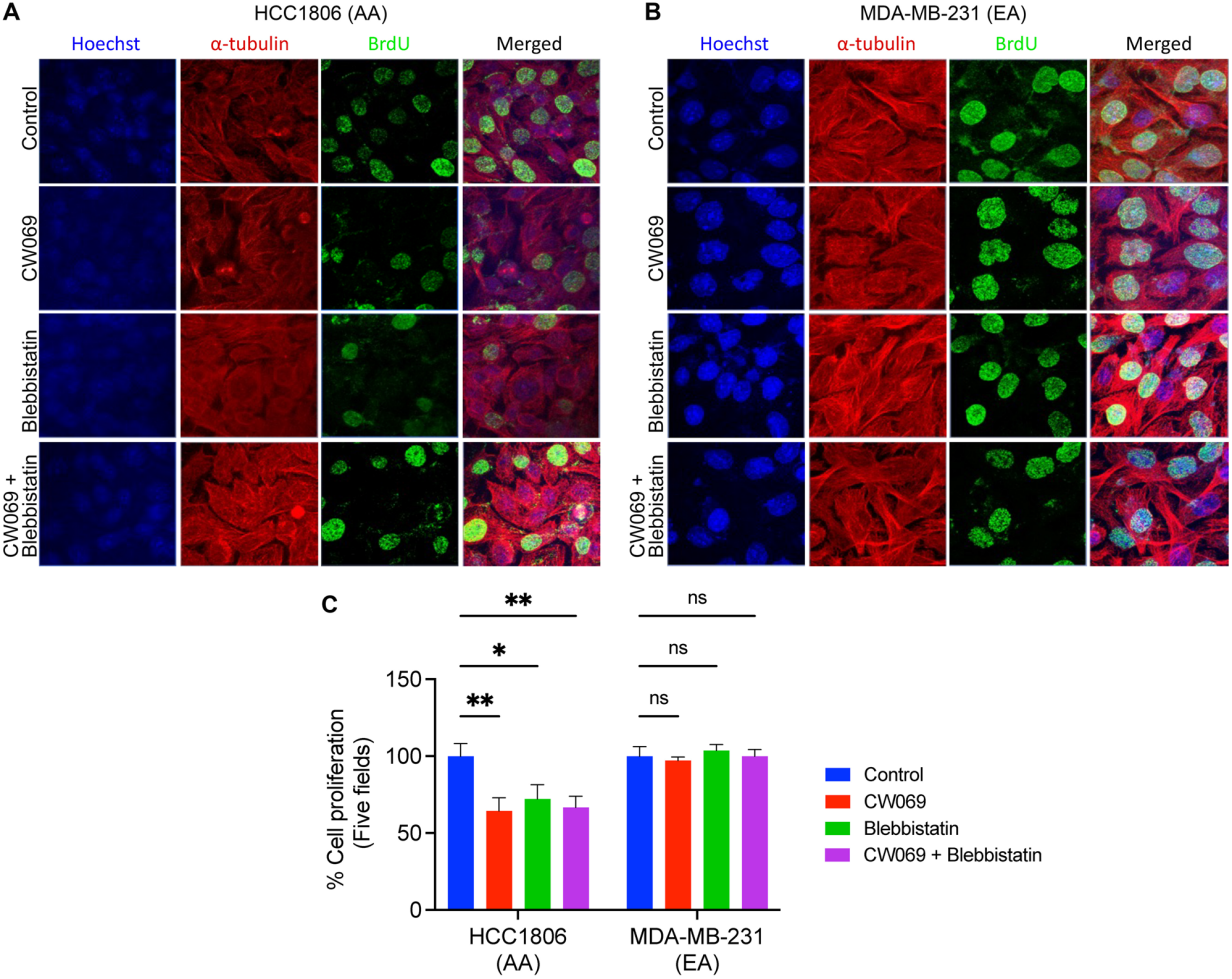
Ablation of KIFC1 and MYH9 attenuates proliferation in AA TNBC cells. Representative IF images (**A, B**) and their quantitative bar graphs (**C**) showing proliferation of AA (**A, C**) and EA (B, C) TNBC cells in control, CW069, blebbistatin, CW069 + blebbistatin-treated AA and EA TNBC cells. *P < 0.05, **P < 0.005

KIFC1 and MYH9 regulate cancer cell proliferation [18–20]. Since the KIFC1-MYH9 interaction was higher in AA TNBC cells, we hypothesized that this interaction might be crucial for cell proliferation in AA TNBC cells. To test this, we performed BrdU cell proliferation assay in untreated and treated (CW069, blebbistatin, and their combination) AA and EA TNBC cells. Cell proliferation was significantly reduced (~ 1.5-fold) in CW069 + blebbistatin-treated AA TNBC cells but not in EA TNBC cells (Fig. 3A—C). The reduction in proliferation in AA upon ablation of both KIFC1 and MYH9 indicates that KIFC1-MYH9 interaction could be crucial for cell proliferation in AA TNBC cells. MYH9 is a transcription factor and, hence, difficult to target clinically. Thus, targeting KIFC1 alone to disrupt the KIFC1-MYH9 interaction could be a more viable alternative than targeting both proteins.

### KIFC1 KO dysregulates expression of genes related to proliferation, invasion, migration, and metastasis in AA and EA TNBC cells

Using the CRISPR-CAS9 KO approach, we knocked out KIFC1 expression in TNBC cells. KIFC1 KO clones were validated by DNA sequencing (Suppl. Figure 1 A), and sequence alignment revealed the presence of an indel mutation, in which a thymine (T) was inserted into the genomic DNA (Suppl. Figure 1B–C). This indel created a frameshift mutation, generating a premature stop codon in the reading frame at the 52nd base (18th amino acid) (Suppl. Figure 1D). The mutation was confirmed by reverse translation of the sequence using the Sequence Manipulation Suite (Suppl. Figure 1D). KO efficiency (homozygous or heterozygous) was confirmed using western blotting (Suppl. Figure 1E). In homozygous KIFC1 KO cell lines, the efficiency of the manipulation was nearly 100%. These KIFC1 KO cell clones were analyzed by RNA sequencing (RNA-seq) to identify downstream KIFC1 partners contributing to racial disparities in TNBC. RNA-seq of HCC1806, MDA-MB-468, MDA-MB-231, and BT-549 KIFC1 KO cell lines was performed using the Illumina HiSeq platform. Differentially expressed genes (DEGs) were subjected to Ingenuity Pathway Analysis (IPA; Suppl. Figure 2 A-D and Suppl. Figure 3 A, B).

**Fig. 4:**
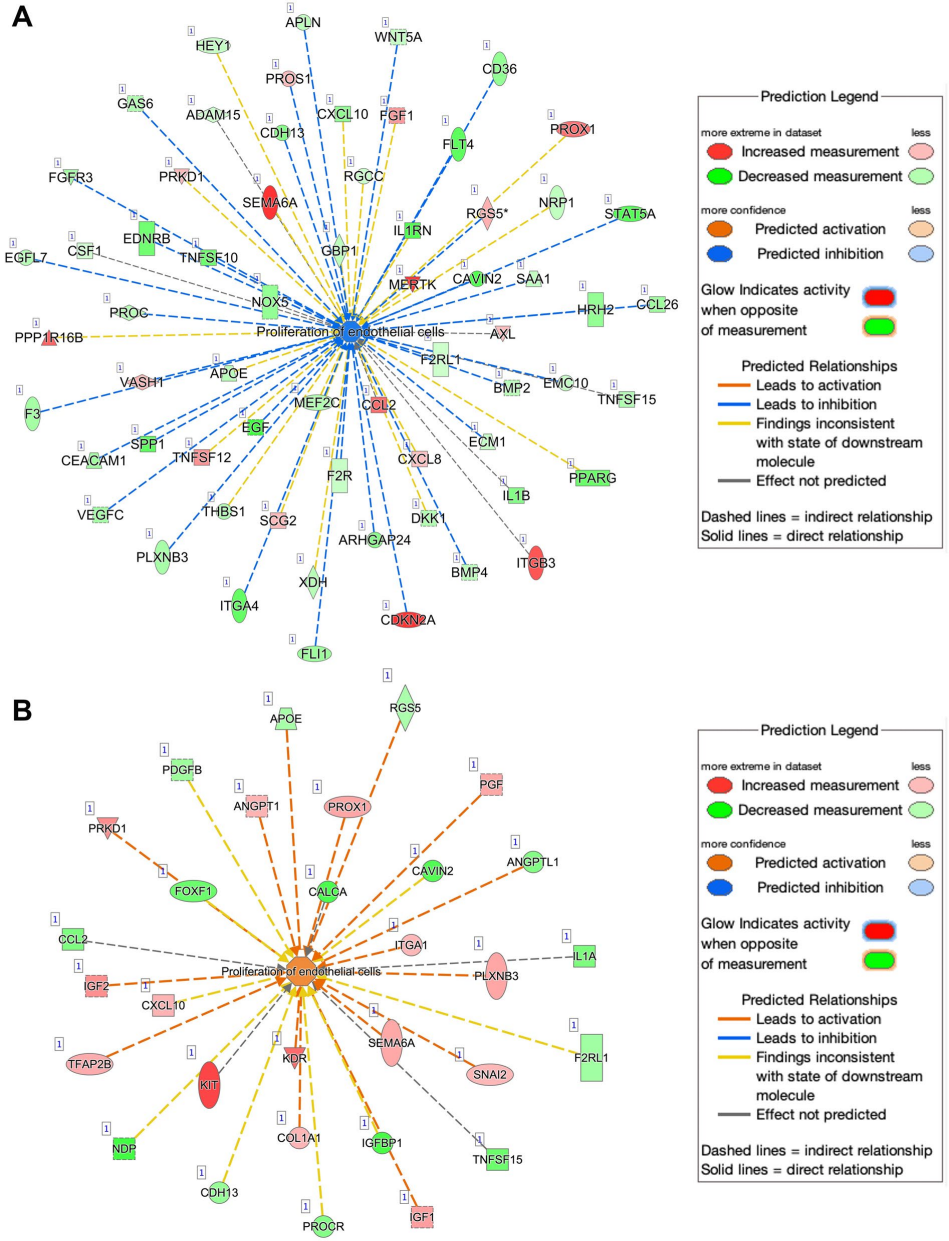
Ingenuity pathway analysis of differentially expressed genes related to proliferation in KIFC1 KO AA (**A**) and EA (**B**) TNBC cells. Inhibition of activity is represented by blue lines; red lines represent activation; yellow lines represent inconsistent findings. Green shapes indicate downregulated genes, whereas red shapes indicate upregulated genes. Solid lines indicate direct connections while dotted lines indicate indirect connections between the genes. Blue octagonal boxes or cross symbols denote downregulated activity. All genes were significantly dysregulated at *P* < 0.05

**Fig. 5:**
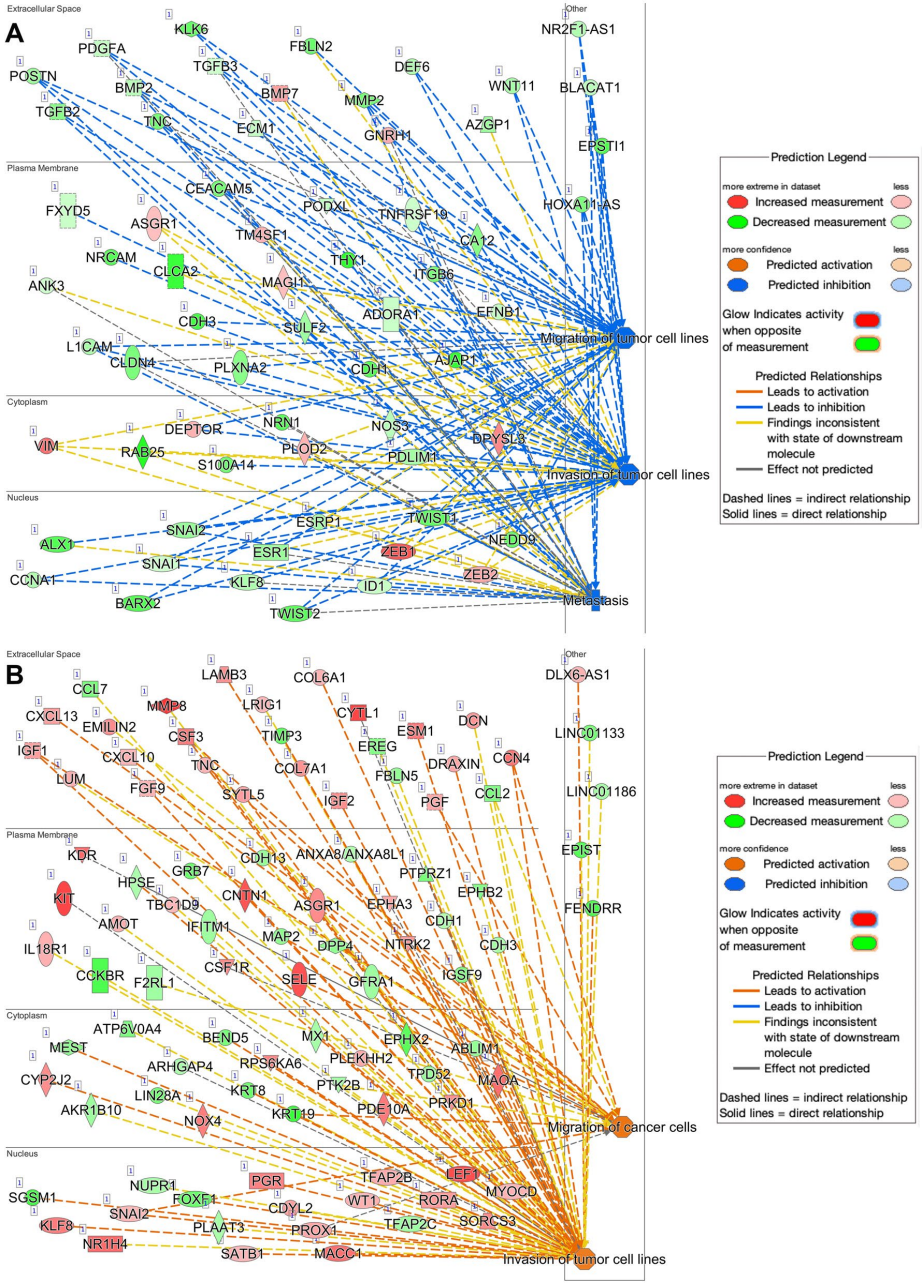
Ingenuity pathway analysis-predicted regulatory effect of differentially expressed genes related to invasion, migration, and metastasis in AA (**A**) and EA (**B**) KIFC1 KO cells. Inhibition of activity is represented by a blue line, whereas the yellow lines represent inconsistent findings. Green shapes indicate downregulated genes, whereas red shapes indicate upregulated genes. Solid lines indicate direct connections while dotted lines indicate indirect connections between the genes. Blue octagonal boxes or cross symbols denote a downregulated activity (migration, cell movement, invasion, and metastasis)

Various genes related to proliferation, invasion, migration, and metastasis were downregulated to a greater extent in AA KIFC1 KO cells than in EA KIFC1 KO cells (Figs. 4 and 5; see details in the Excel workbook). Comparison of KIFC1 KO AA and EA samples revealed significant downregulation of various pathways related to cell movement, migration, invasion, and metastasis in AA KIFC1 KO samples. This finding strengthens our hypothesis that AA TNBC cells rely on KIFC1 for migration and invasion. Gene set enrichment analysis (GSEA) of TNBC patient samples revealed that Wnt/b-catenin signaling, a pathway crucial for metastasis, was upregulated in AA TNBC cells compared with EA TNBC cells (Suppl. Figure 4 A). Moreover, MYC/c-MYC, an important gene for metastasis and downstream effector of the Wnt/b-catenin pathway, was expressed at higher levels in AA vs. EA TNBC samples in the TCGA dataset (Suppl. Figure 4B).

Network analysis (using the grow function of IPA) of DEGs between AA and EA KIFC1 KO samples confirmed that many genes under-expressed in AA TNBC cells were related to the inhibition of migration, invasion, and metastasis (Fig. 5A, B; see details in the Excel workbook). Significant pathways were used for further analysis. Consistent with our previous findings, AA KIFC1 KO TNBC cells exhibited profound dysregulation in various pathways related to cell movement, invasion, and migration (detailed Excel workbooks with genes and expression are provided in the supplementary data). The regulatory effect functional relationship of this analysis showed that approximately 40% of genes (12 of the 30 genes) are involved in metastasis (Fig. 5A, B). Furthermore, proliferation-related genes were downregulated more strongly in AA KIFC1 KO cells than in EA KIFC1 KO cells.

**Fig. 6:**
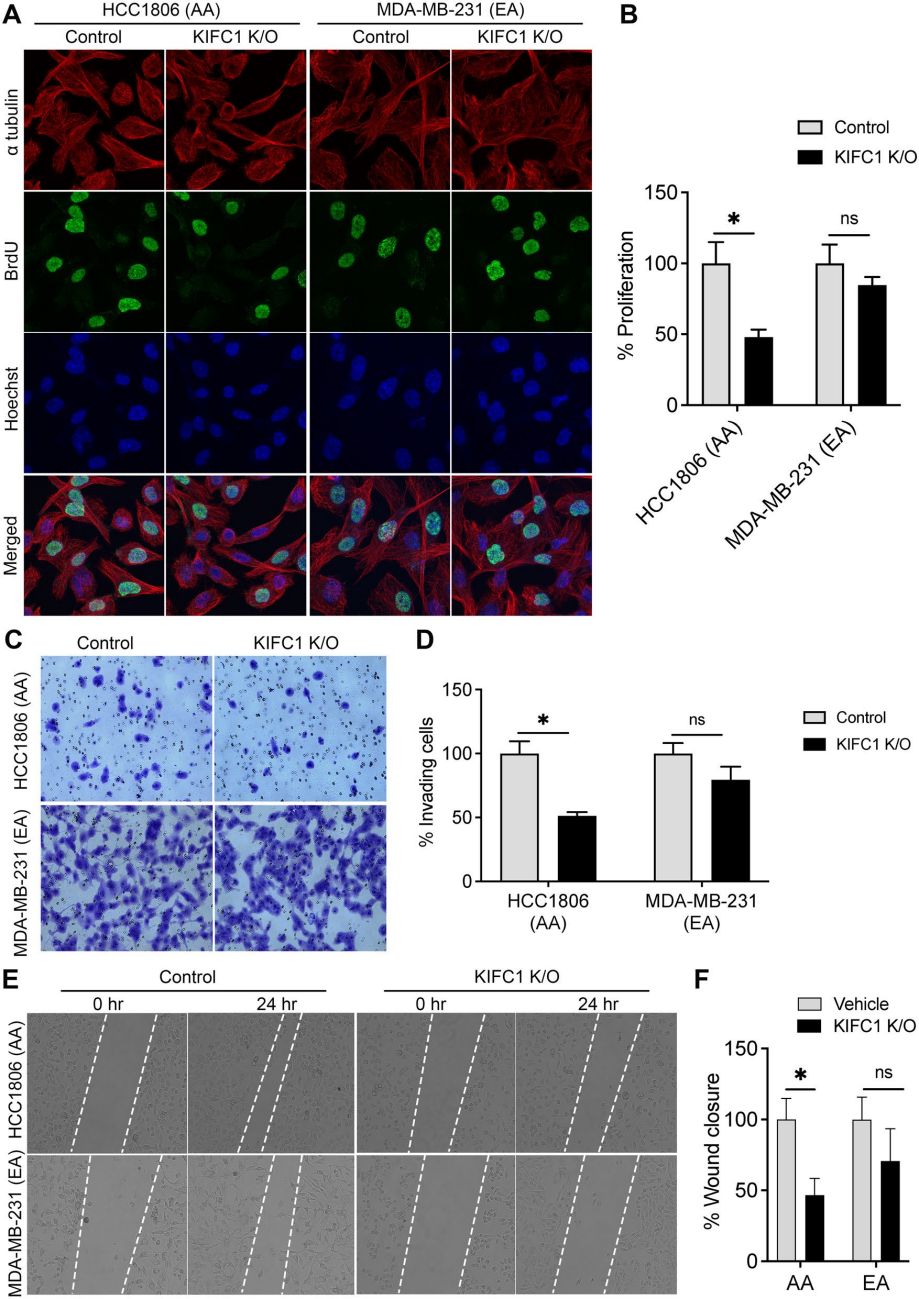
Representative immunofluorescence images (**A**) and bar graphs (**B**) showing BrdU staining in control and KIFC1 KO AA and EA TNBC cell lines. (**C**, **D**) Representative images (**C**) and bar graphs (**D**) showing invading control and KIFC1 KO AA and EA TNBC cells stained with crystal violet. Representative images (**E**) and bar graphs (**F**) showing wound closure in control and KIFC1 KO AA and EA TNBC cells. ns-non-significant, **-*p* < 0.005

Next, we performed functional assays to validate the RNA-seq data. KIFC1 KO TNBC cells were used to investigate the role of KIFC1 in TNBC cell proliferation, migration, and invasion. Immunofluorescence and spectrophotometry analyses revealed that BrdU incorporation was significantly lower in AA TNBC KIFC1 KO cells than in EA TNBC KIFC1 KO (Fig. 6A, B), suggesting that AA TNBC cells are more dependent on KIFC1 for cell proliferation than EA TNBC cells. Furthermore, KIFC1 KO significantly inhibited cell invasion (Fig. 6C, D) and migration (Fig. 6E, F) in AA TNBC cells. These results suggest that KIFC1 may contribute to the high aggressiveness of TNBC in AA patients by enhancing TNBC cell proliferation, invasion, and migration. Additionally, these functional data confirm the findings from RNA-seq analyses predicting a more critical role for KIFC1 in TNBC cell migration and invasion in AAs than in EAs.

**Fig. 7:**
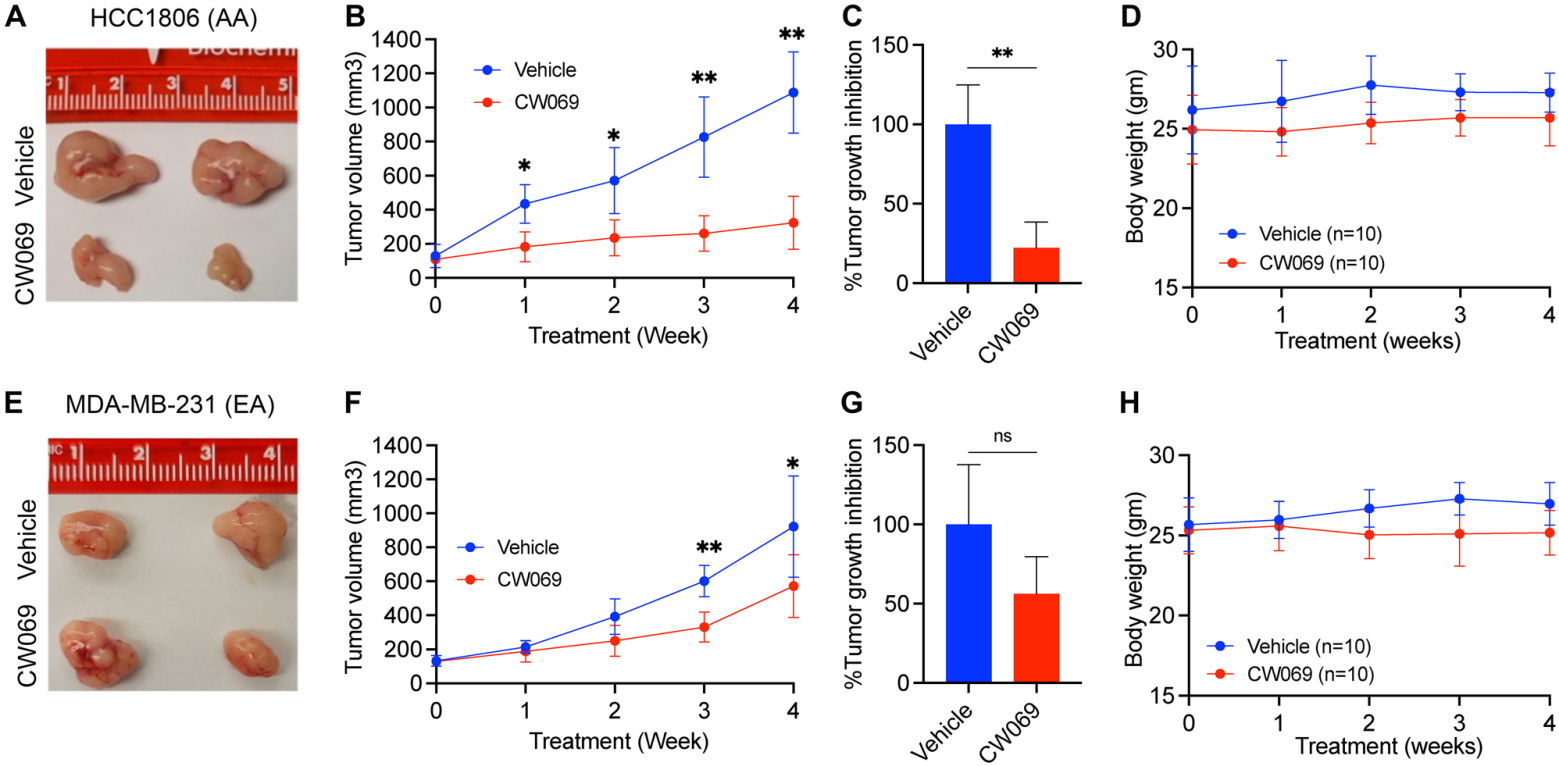
Representative tumor images (**A**, **E**) and quantitative bar graphs (**B**, **F**) showing tumor volume in vehicle- and CW069-treated mice bearing AA and EA TNBC tumors. (**C**, **G**) Bar graphs showing % tumor proliferation in vehicle- and CW069-treated mice bearing AA and EA TNBC. (**D**, **H**) Graphs showing body weights of vehicle and CW069 treated mice bearing AA and EA TNBC cells. *P < 0.05, **P < 0.005; ns, non-significant

### CW069 significantly reduces proliferation in mice bearing AA TNBC xenografts

To validate our in vitro data, we utilized xenograft mice models bearing AA and EA TNBC cells. We did not observe any differences in the body weight of mice between the treatment groups (Fig. 7D, H), suggesting an excellent safety profile for the CW069 regimens. We assessed the effect of CW069 on tumor growth inhibition in AA and EA TNBC xenografts. We observed a significant tumor growth inhibition in AA compared to EA TNBC xenografts (Fig. 7A–C, E–G).

These results align with our in vitro data and suggest that inhibition of KIFC1 is linked to reduced tumor growth in AA TNBC, possibly due to disruption of the interaction between KIFC1 and MYH9. These findings support that targeting KIFC1 using CW069 could represent a promising therapeutic strategy for AA patients with TNBC.

## Discussion

TNBC patients of African descent, especially those with advanced disease at diagnosis, have a worse prognosis than patients of European descent [15, 21–24]. TNBC patients, many of whom are AA, are left with very few treatment options due to the lack of therapeutic targets, including ER, PR, and HER2. In addition, standard anticancer therapies do not benefit AA patients with TNBC as much as they do other patient groups. As AA women with TNBC typically exhibit a much more aggressive disease course than EA women, elucidating the mechanisms underlying racial disparities in TNBC is of high clinical importance.

We have previously shown that nKIFC1 is associated with poor survival outcomes in AA but not in EA patients with TNBC. Notably, nKIFC1 significantly predicted survival outcomes in AA but not in EA patients with TNBC. We have shown that high nKIFC1 weighted index was significantly associated with poor overall survival, progression-free survival, and distant metastasis-free survival (hazard ratio [HR] = 3.5, 3.1, and 3.8, respectively; *P* = 0.01, 0.009, and 0.007, respectively) in multivariable Cox proportional-hazards models in AA but not in EA patients with TNBCs [18]. The varied prognostic value of nKIFC1 in these two racially distinct groups may stem from inherent differences in tumor biology rather than the substantially higher basal nKIFC1 levels in TNBCs from AAs than in those from EAs.

It is well established that protein-protein interactions, rather than individual proteins alone, regulate neoplastic progression. The tumor-promoting function of several aberrantly expressed proteins in the cancerous state is directly proportional to their ability to interact with a protein-binding partner [25]. In the present study, we found higher MYH9 abundance with nKIFC1 in TNBC cells from AAs than in those from EAs, which may provide mechanistic insights into the dependency of AA TNBC cells on KIFC1 for invasion and migration. MYH9, a member of the myosin II subfamily [26], plays an important role in cell adhesion, cytokinesis, and maintenance of cell morphology [27]. MYH9 has been reported to play a crucial role in cancer cell proliferation, survival, invasion, and metastasis [28]. There are conflicting results regarding the role of MYH9 as a tumor suppressor or oncogene. In BC, several studies have shed light on the roles of MYH9 in tumor metastasis [29, 30]. A recent study by Li et al. [31] showed that the interaction between EIF6-224aa and MYH9 activates the Wnt/β-catenin pathway and promotes the proliferation and metastasis of TNBC cells. Our DEGs and IPA data also suggest that the nKIFC1-MYH9 complex may regulate the expression of the Wnt pathway, EMT, and other genes involved in invasiveness. KIFC1 immunoprecipitates with M7CKs, the nuclear-localized cleaved kinase domain of the chanzyme TRPM7, and M7CKs, in turn, are part of a chromatin-modifying complex that also binds to nuclear β-catenin and is implicated in BC [32]. MYH9 also regulates actin-related processes in the cytoplasm and functions as a core transcription factor in the nucleus in concert with nuclear actin. Nuclear actin tethers RNA polymerase II to the nucleoskeleton, thereby facilitating transcription. DDX5 is a co-activator of β-catenin. We showed that racially distinct KIFC1 complexes exist in the nuclei of TNBC cells. The mass spectrometry data and identification of DDX helicases, actin, and actinins appear to be more in line with their role in chromatin remodeling and maintenance, and perhaps even the regulation of gene expression in interphase. KIFC1 can transport bare DNA oligonucleotides along microtubules in HeLa cells. Given that M7CKs bind to β-catenin and KIFC1 in the nucleus, nKIFC1 may amplify Wnt/β-catenin signaling, which was observed in our mass spectrometry data. Based on our data, we suggest that nKIFC1 impairs the tumor suppressor function of MYH9 and facilitates the amplification of Wnt/β-catenin signaling to a greater extent in AA than in EA TNBC cells. KIFC1 and MYH9 could be part of a chromatin remodeling complex that includes the Wnt signaling co-activator DDX5 exclusively in AA, but not in EA TNBCs. This complex may enhance Wnt/β-catenin signaling (to upregulate targets such as MYC) more prominently in AA than in EA TNBCs.

As MYH9 is a transcription factor and essential for normal cells, targeting KIFC1 to inhibit oncogenic KIFC1-MYH9 signaling represents an attractive therapeutic option for AA patients with TNBCs. High nKIFC1 levels in AAs with TNBC seem to play a critical role in tumor cell proliferation, migration, and metastasis, conferring a worse prognosis. Our in vivo data strongly suggest that CW069 could be a novel alternative therapeutic approach for AA patients with TNBC. This study revealed that the nuclear accumulation of KIFC1 is a novel mechanism contributing to racial disparities in TNBC and demonstrated that KIFC1 is crucial for cell proliferation, migration, and invasion in TNBC cells from AAs, but not in those from EAs, opening new avenues for further research. Our results align with those of previous studies suggesting that KIFC1 is crucial for disease aggressiveness and metastasis [18, 19]. Thus, KIFC1 inhibition using commercially available inhibitors, such as CW069, may have a more prominent effect on the inhibition of TNBC metastasis in AAs than in EAs.

Even though the results presented in this study are novel, confirmation of the findings in large patient cohorts is required. Correlating nKIFC1 immunohistochemical staining scores with percent African ancestry could provide additional clues about the potential use of KIFC1 as a therapeutic target for AA patients with TNBC. Because African ancestry is an independent predictor of poor outcomes, the percentage of African ancestry can confound survival analysis. Thus, the AA cohort is likely to be substantially admixed. Therefore, nKIFC1 may merely stratify patients based on the percentage of African ancestry. Because the number of patients with very high African ancestry will only be a fraction of the entire cohort (only ~ 10% of AAs have > 90% African ancestry [33]), the significance of findings may be diminished. Thus, it is important to test the prognostic ability of nKIFC1 in native African populations (> 90% of African ancestry). Demonstrating the prognostic ability of KIFC1 in a minimally admixed AA cohort would help to determine whether it can risk-stratify patients of high African ancestry. Genetic ancestry analyses of US and African cohorts are currently underway. Moreover, the ability of CW069 to inhibit tumor growth and metastasis in AA and EA TNBC xenograft mouse models requires further investigation. These results may provide further preclinical evidence on the efficacy of CW069 in targeting KIFC1 in AA patients with TNBC.

### Electronic supplementary material

Below is the link to the electronic supplementary material.

Supplementary Material 1

Supplementary Material 2

## Data Availability

The data of this study will be shared by the corresponding author (R.A.) upon reasonable request.
