# Supplementary material for: A novel role for KIFC1-MYH9 interaction in triple-negative breast cancer aggressiveness and racial disparity
Source: Cell Commun Signal. 2024 Jun 6;22:312. doi: 10.1186/s12964-024-01664-0 (PMC11188183; doi:10.1186/s12964-024-01664-0)

Raw blots

Figure-1 (C) IB of KIFC1 IP samples used for MS, input blots of MYH9, KIFC1 and beta-actin


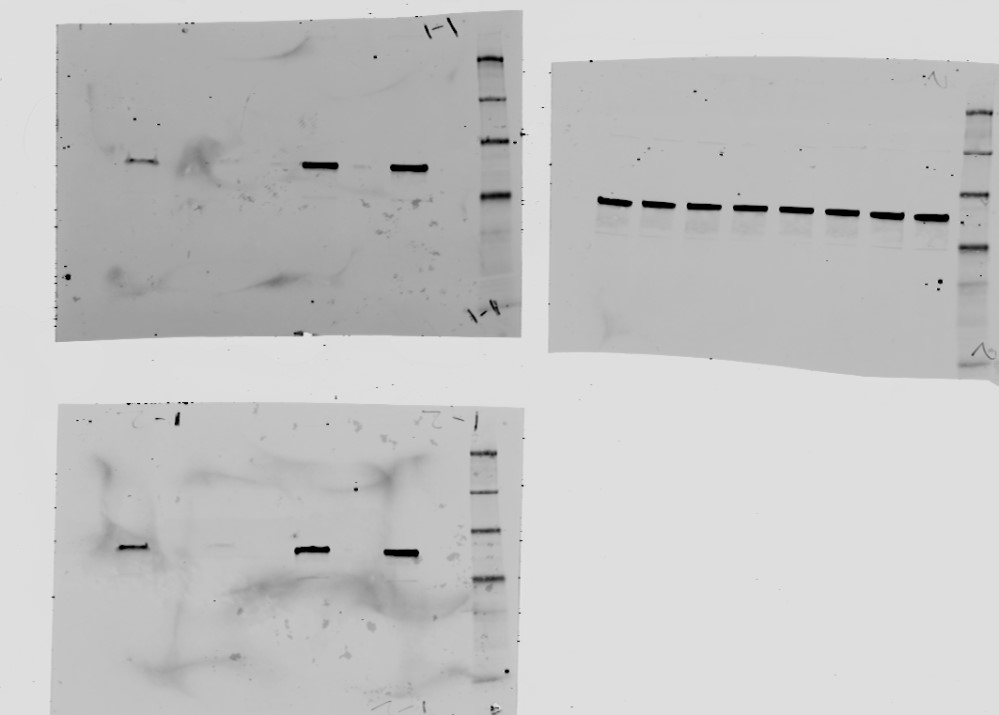


IP: IB: MYH9


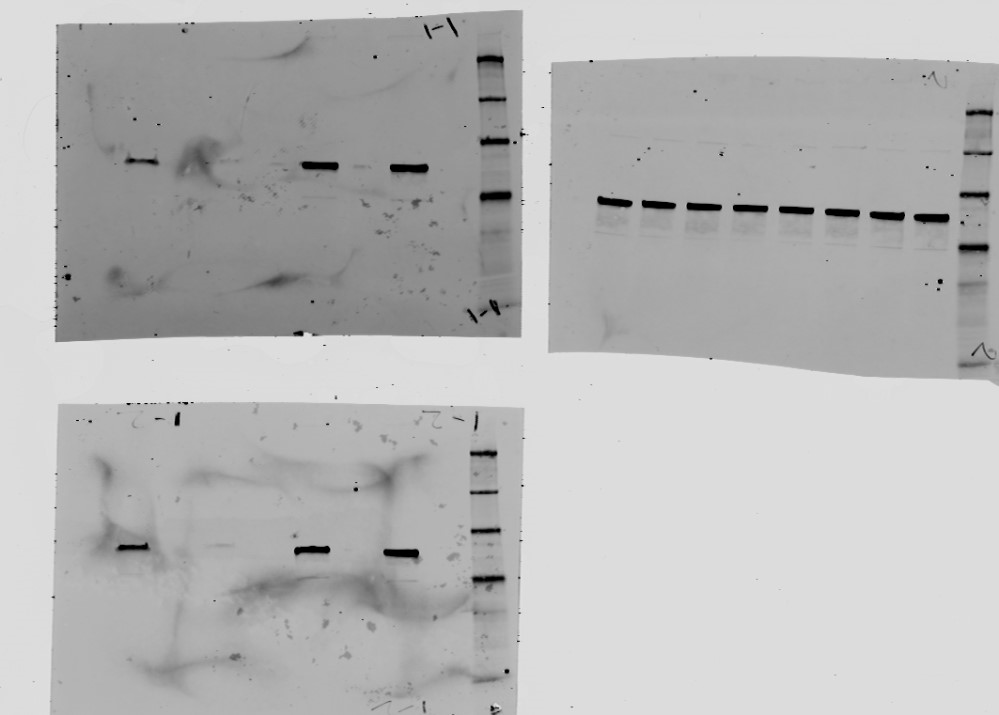


MYH9 input Blot


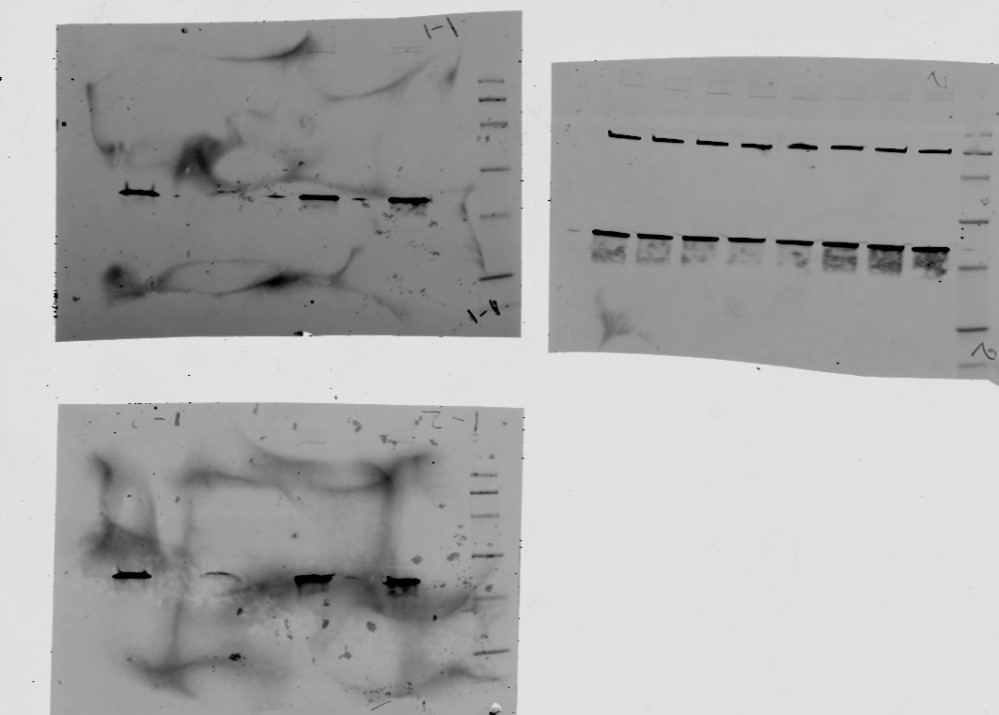


KIFC1 input blot


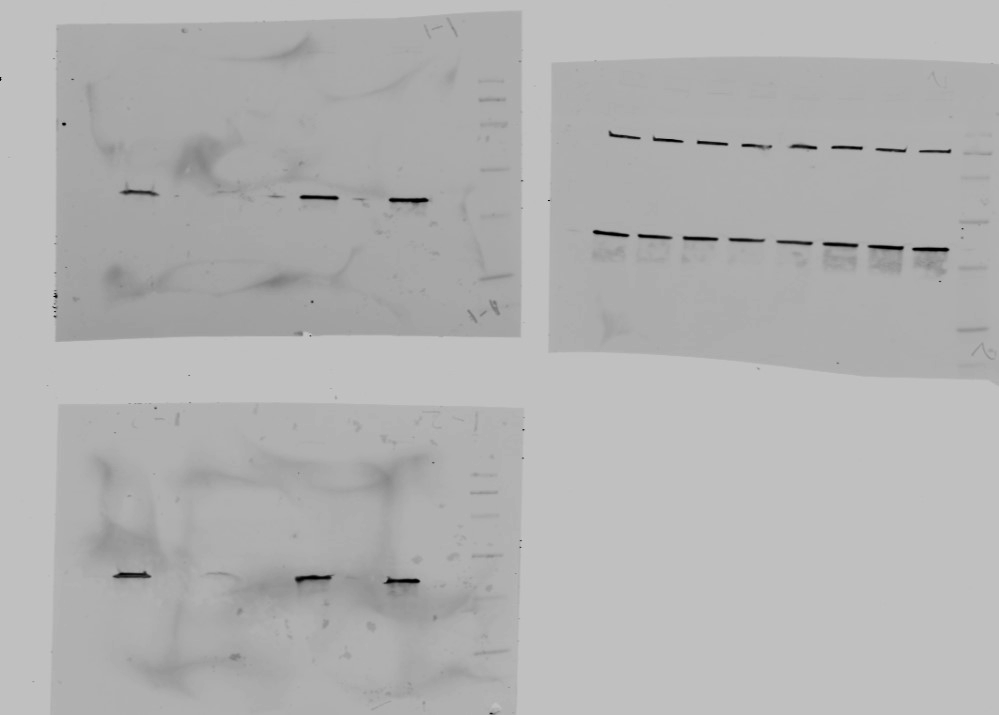


HDAC-2 input blot

Figure-2 (C) Knockdown (KD) blots of MYH9, KIFC1 with beta-actin


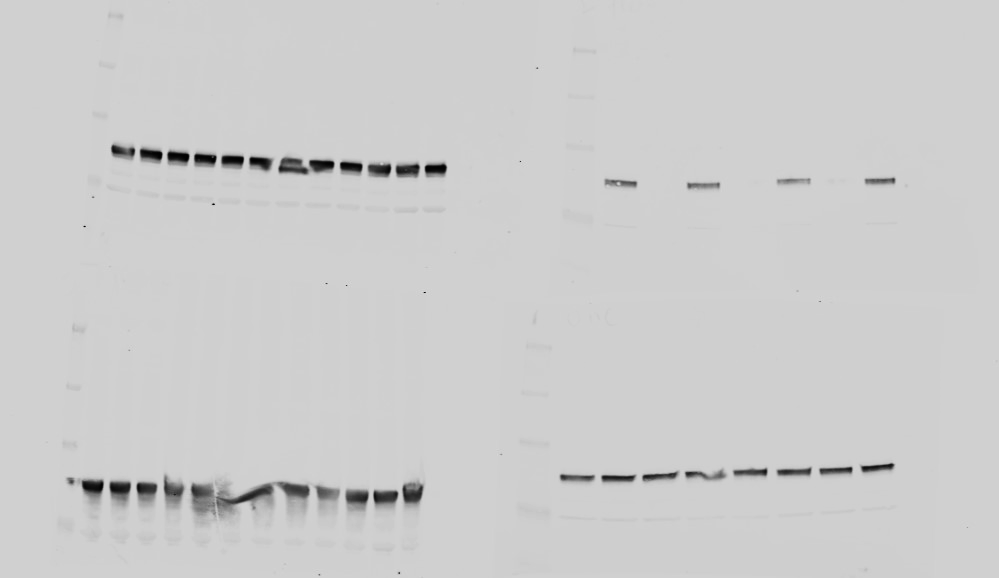


MYH9 KD


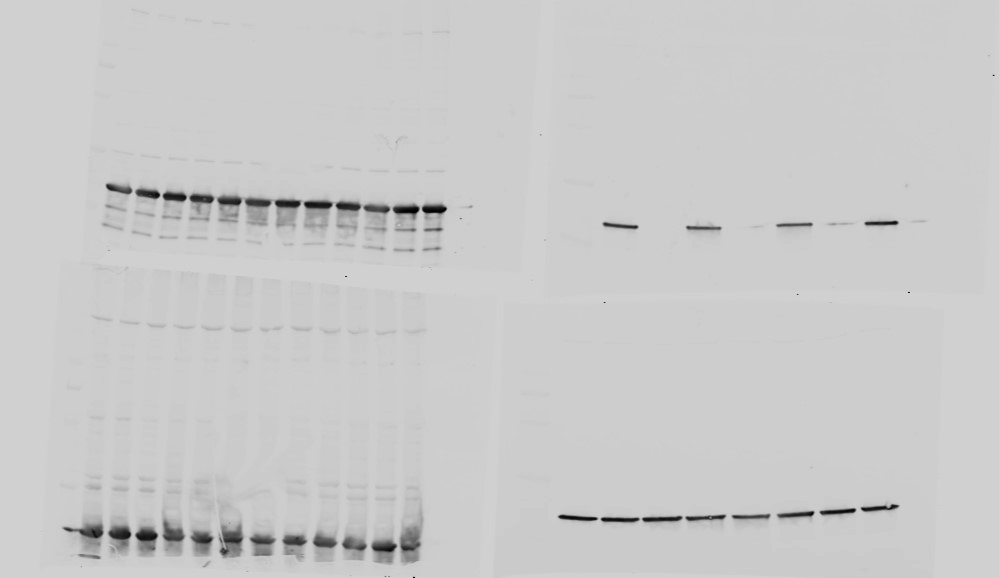


KIFC1 KD


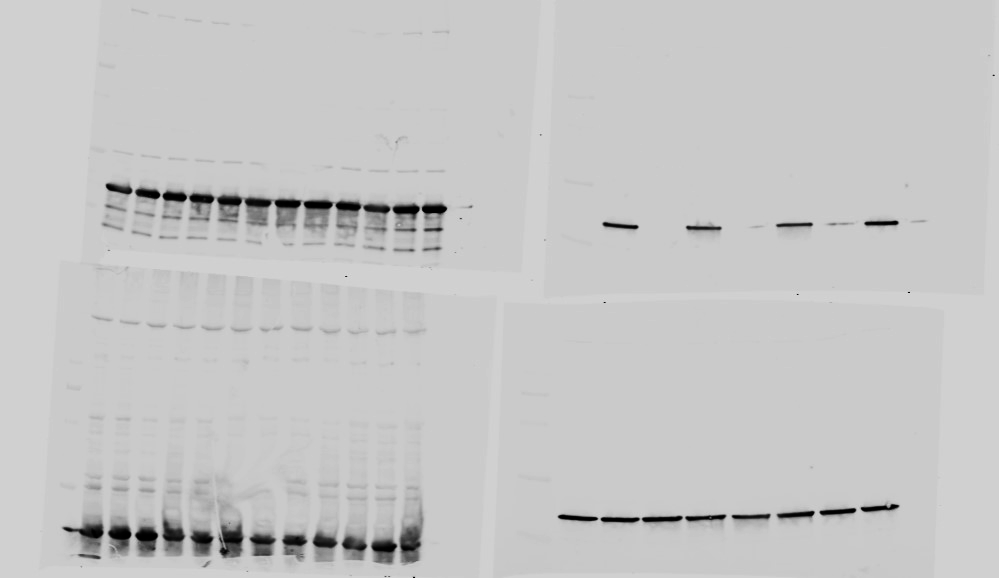


B actin

Raw blots of Supplementary figure-1 (E)

KIFC1 blot containing AA and EA WT and KO cell lysates.


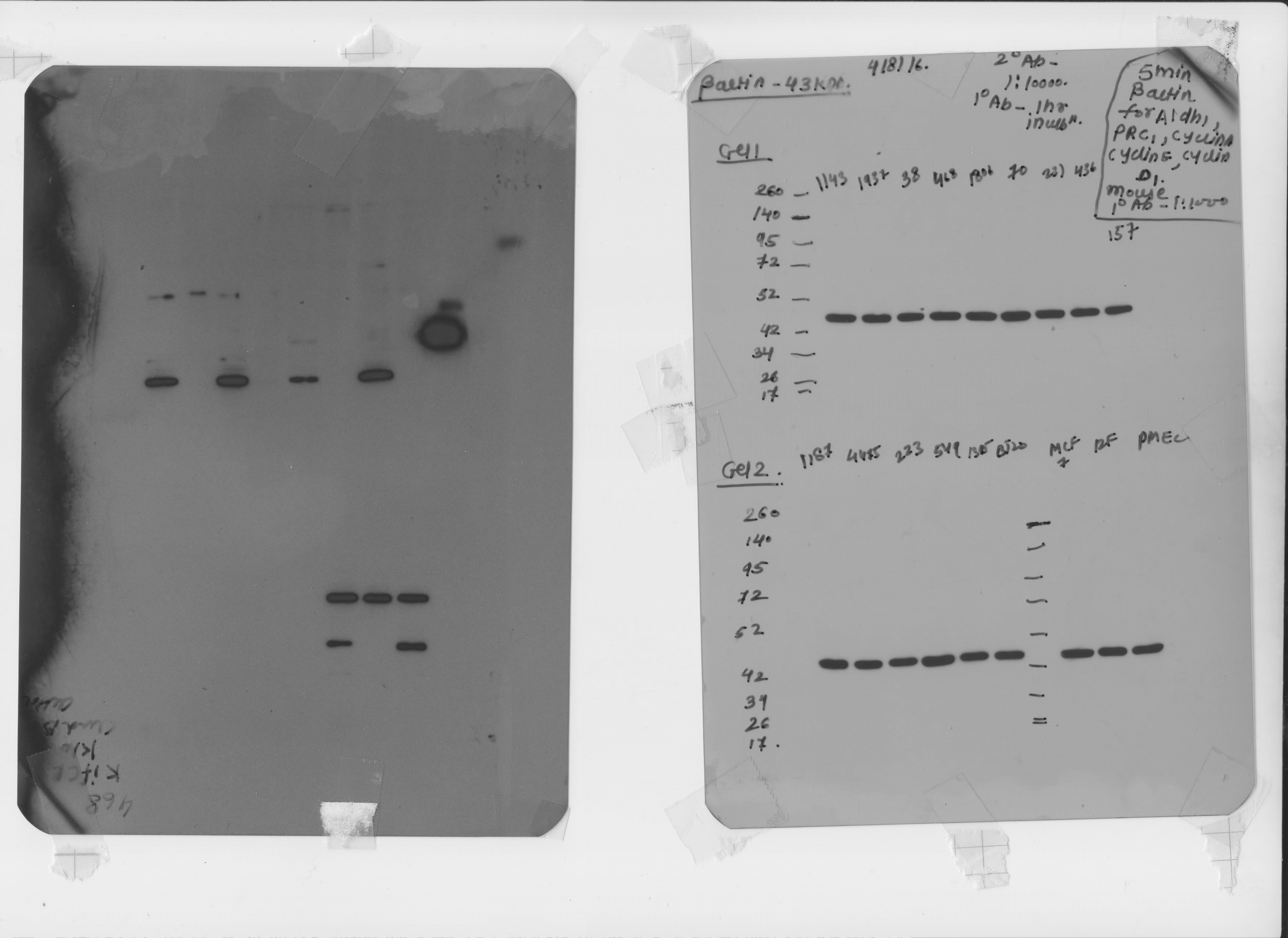


β-Actin blot containing AA and EA WT and KO cell lysates.


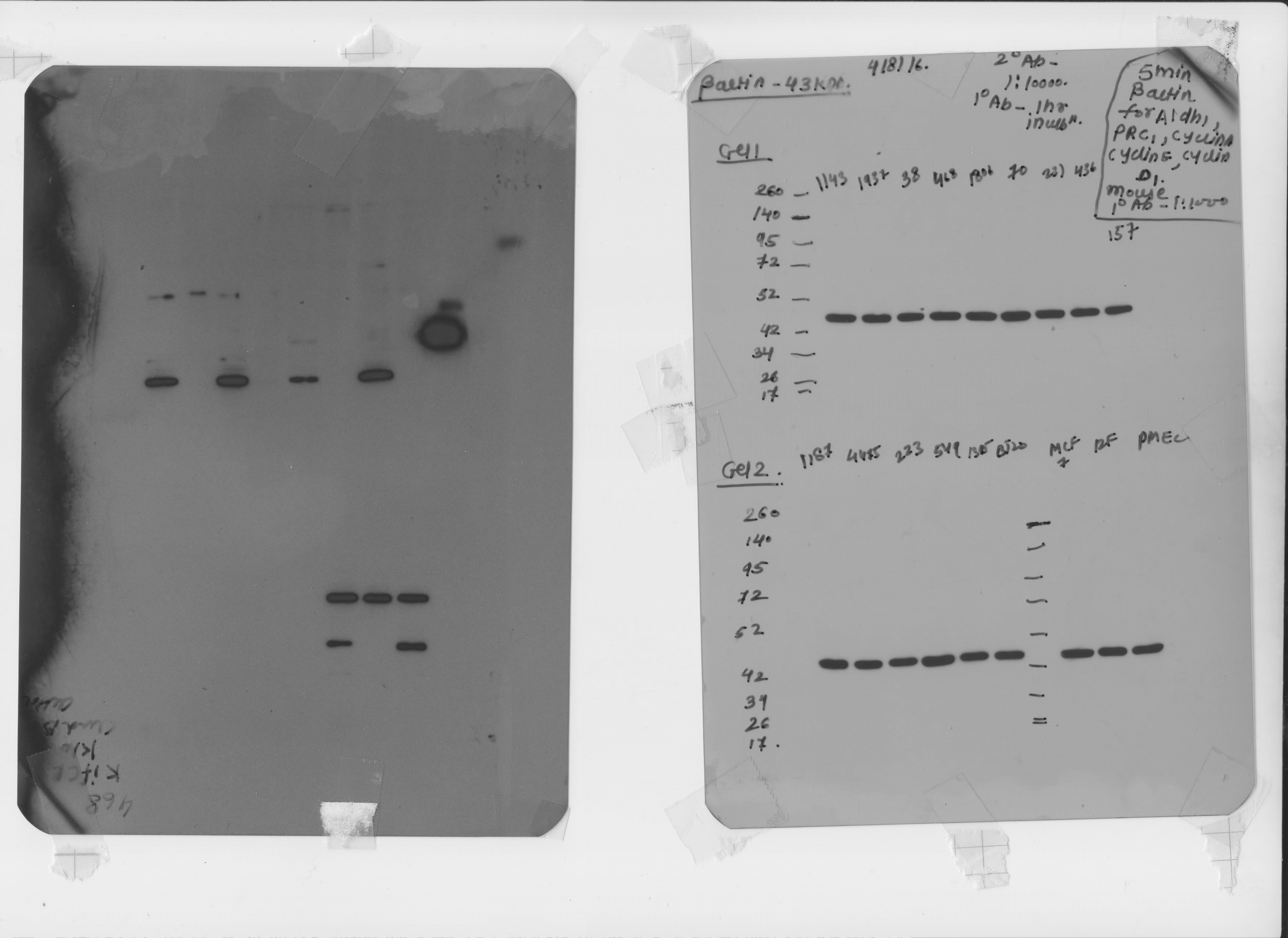

Supplement: Supplementary file 1 — Supplementary Material 1 [file 12964_2024_1664_MOESM1_ESM.docx]
