# Supplementary material for: A novel role for KIFC1-MYH9 interaction in triple-negative breast cancer aggressiveness and racial disparity
Source: Cell Commun Signal. 2024 Jun 6;22:312. doi: 10.1186/s12964-024-01664-0 (PMC11188183; doi:10.1186/s12964-024-01664-0)
Supplement: Supplementary file 2 — Supplementary Material 2 [file 12964_2024_1664_MOESM2_ESM.docx]

**Supplementary figures**


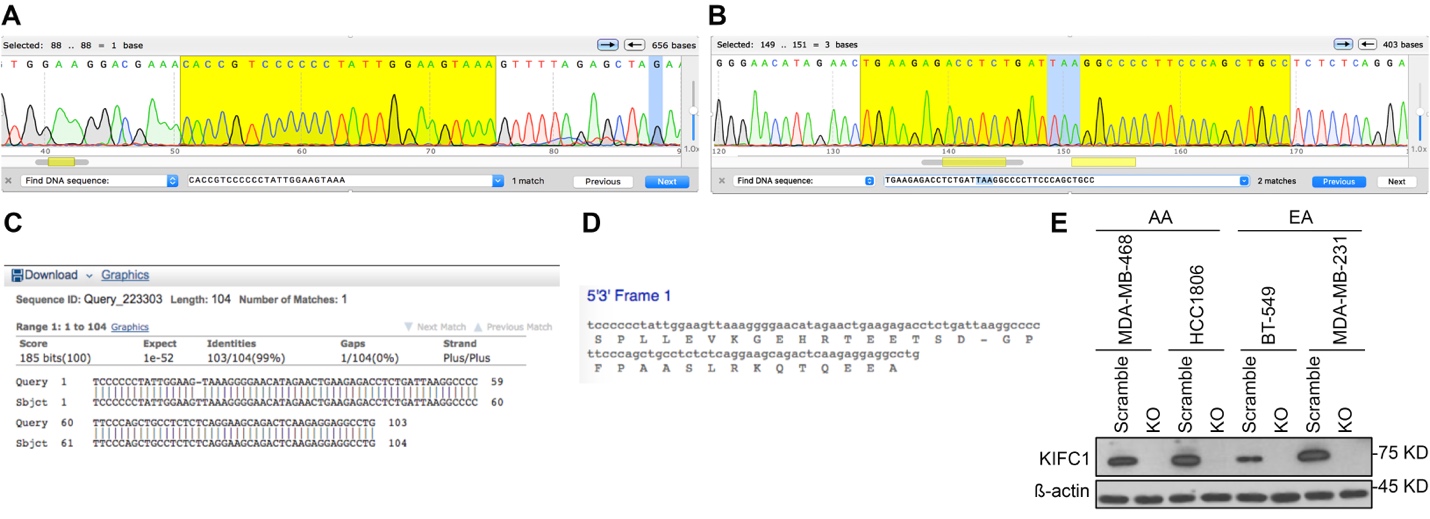


Supplementary figure 1: Confirmation of KIFC1 KO in TNBC cells. (A, B) DNA sequence histogram. Mutations in KIFC1 in TNBC cell lines from AA and EA patients are highlighted. (C, D) NCBI sequence alignment for KIFC1 showing a point mutation (C) and insertion of a stop codon (D) in KIFC1 KO cells. (E) Western blot showing KIFC1 depletion in AA and EA TNBC cell lines. Representative homozygous KIFC1 KO samples are shown. Β-actin was used as a loading control.


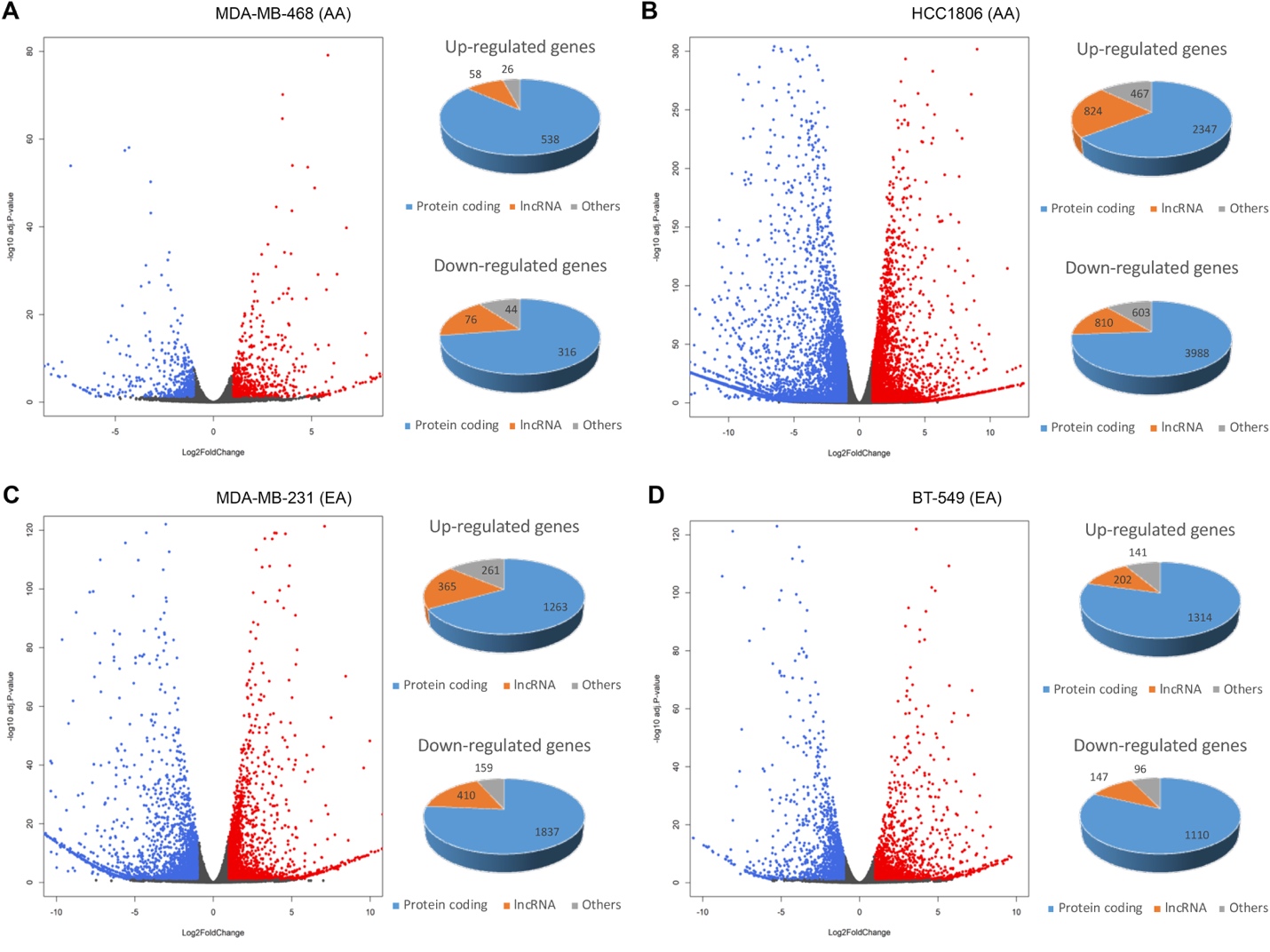


Supp figure 2. Ingenuity pathway analysis showing dot plots and pie charts of differentially expressed RNAs in KIFC1 KO AA (A-B) and EA (C-D) TNBC cells. All genes were significantly dysregulated at P > 0.05. A: HCC1806, B: MDA-MB-468, C: BT549, D: MDA-MB-231. Red dot plots indicate upregulated RNAs while blue dot plots indicate downregulated RNAs


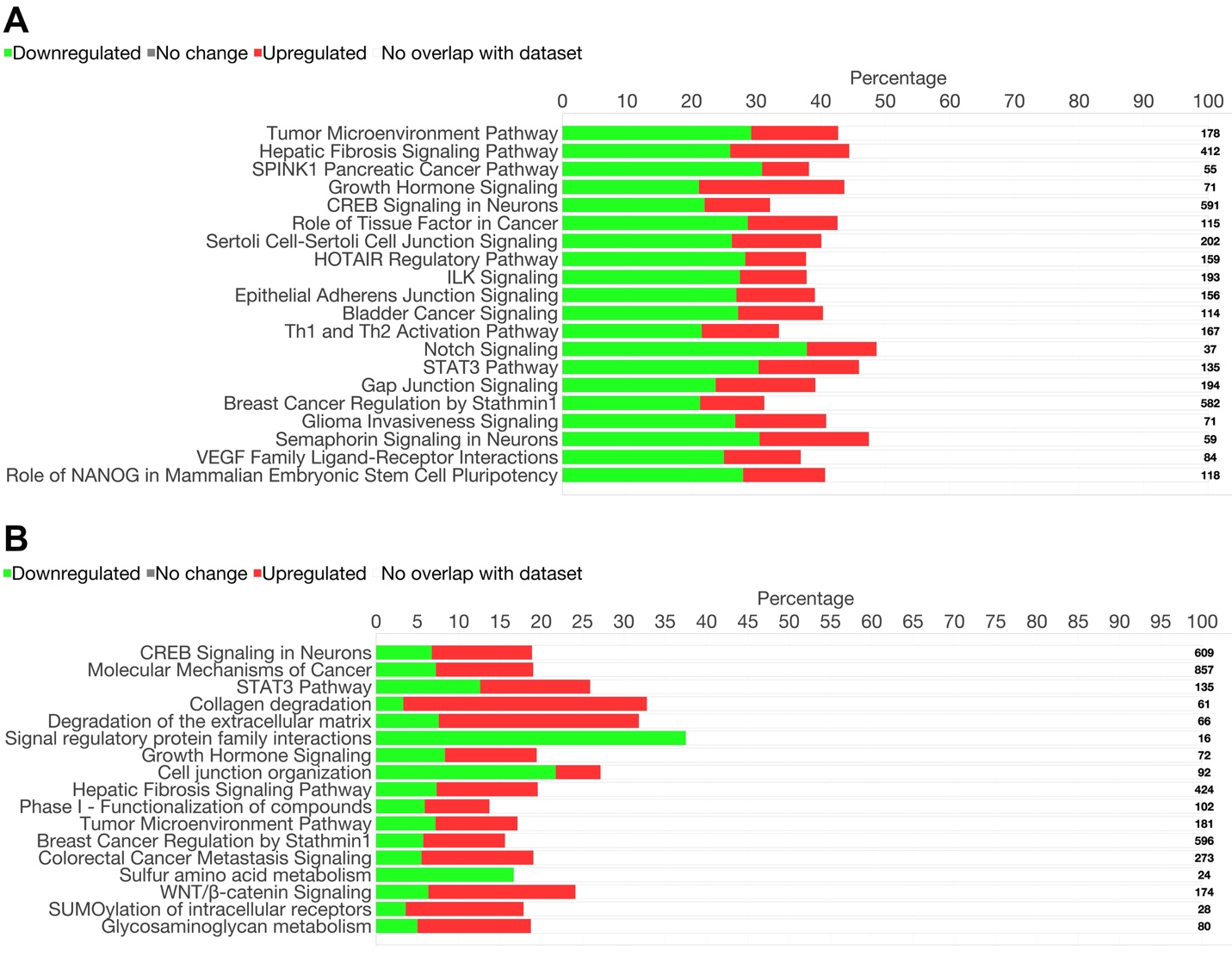


Supplementary figure 3 Ingenuity pathway analysis of differentially expressed genes in KIFC1 KO AA (A) and EA (B) TNBC cells. All genes were significantly dysregulated at P > 0.05.


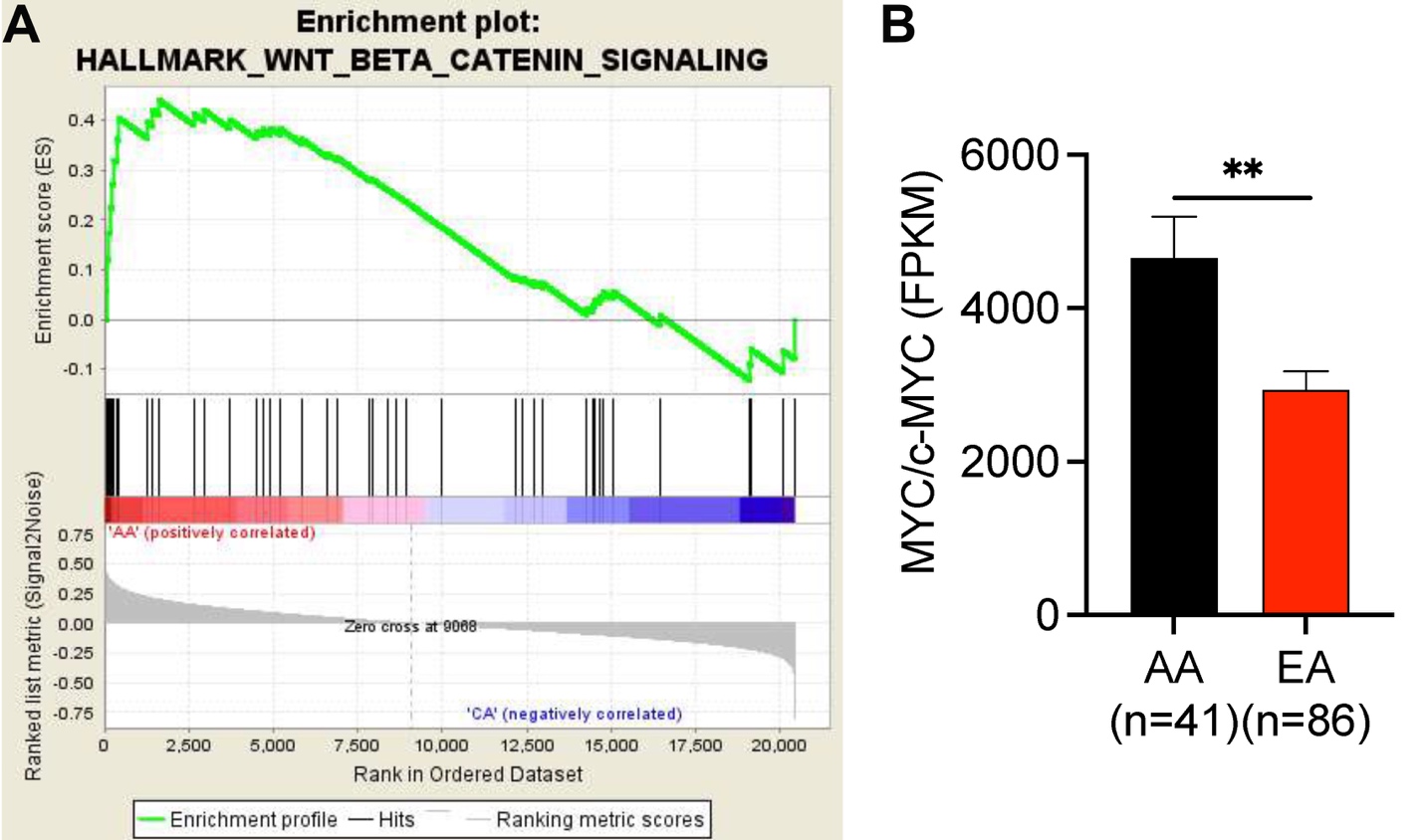


Supp fig. 4. GSEA (A) and TCGA (B) data showing upregulation of metastatic genes such as WNT β-catenin and MYC in TNBC patients
